# Molecular patterns of cancer colonisation in lymph nodes of breast cancer patients

**DOI:** 10.1186/s13058-018-1070-3

**Published:** 2018-11-20

**Authors:** Gaurav Chatterjee, Trupti Pai, Thomas Hardiman, Kelly Avery-Kiejda, Rodney J. Scott, Jo Spencer, Sarah E. Pinder, Anita Grigoriadis

**Affiliations:** 1 Cancer Bioinformatics, King’s College London, Innovation Hub, Cancer Centre at Guy’s Hospital, Great Maze Pond, London, SE1 9RT UK; 2 School of Cancer & Pharmaceutical Sciences, CRUK King’s Health Partners Centre, King’s College London, Innovation Hub, Comprehensive Cancer Centre at Guy’s Hospital, Great Maze Pond, London, SE1 9RT UK; 3 Department of Pathology, Tata Memorial Centre, 8th Floor, Annexe Building, Mumbai, India; 4 Priority Research Centre for Cancer, School of Biomedical Sciences and Pharmacy, Faculty of Health, University of Newcastle, Newcastle, NSW 2308 Australia; 5 Peter Gorer Department of Immunobiology, King’s College London, Guy’s Hospital, 2nd Floor, Borough Wing, London, SE1 9RT UK; 6 Breast Cancer Now Research Unit, Innovation Hub, Cancer Centre at Guy’s Hospital, King’s College London, Faculty of Life Sciences and Medicine, London, SE1 9RT UK

**Keywords:** Expression, Lymph node, Premetastatic niche, Breast cancer

## Abstract

Lymph node (LN) metastasis is an important prognostic parameter in breast carcinoma, a crucial site for tumour–immune cell interaction and a gateway for further dissemination of tumour cells to other metastatic sites. To gain insight into the underlying molecular changes from the pre-metastatic, via initial colonisation to the fully involved LN, we reviewed transcriptional research along the evolving microenvironment of LNs in human breast cancers patients. Gene expression studies were compiled and subjected to pathway-based analyses, with an emphasis on immune cell-related genes. Of 366 studies, 14 performed genome-wide gene expression comparisons and were divided into six clinical-biological scenarios capturing different stages of the metastatic pathway in the LN, as follows: metastatically involved LNs are compared to their patient-matched primary breast carcinomas (scenario 1) or the normal breast tissue (scenario 2). In scenario 3, uninvolved LNs were compared between LN-positive patients and LN-negative patients. Scenario 4 homed in on the residual uninvolved portion of involved LNs and compared it to the patient-matched uninvolved LNs. Scenario 5 contrasted uninvolved and involved LNs, whilst in scenario 6 involved (sentinel) LNs were assessed between patients with other either positive or negative LNs (non-sentinel).

Gene lists from these chronological steps of LN metastasis indicated that gene patterns reflecting deficiencies in dendritic cells and hyper-proliferation of B cells parallel to tumour promoting pathways, including cell adhesion, extracellular matrix remodelling, cell motility and DNA repair, play key roles in the changing microenvironment of a pro-metastatic to a metastatically involved LN. Similarities between uninvolved LNs and the residual uninvolved portion of involved LNs hinted that LN alterations expose systemic tumour-related immune responses in breast cancer patients. Despite the diverse settings, gene expression patterns at different stages of metastatic colonisation in LNs were recognised and may provide potential avenues for clinical interventions to counteract disease progression for breast cancer patients.

**Electronic supplementary material:**

The online version of this article (10.1186/s13058-018-1070-3) contains supplementary material, which is available to authorized users.

## Introduction

The lymph nodes (LNs) are functional units of the immune system that act as immunological hubs supporting the complex interactions between T cells, B cells, antigen-presenting cells and stromal cells. LNs receive cells and potential immunogenic substances via the afferent lymphatics that drain the tissues and enter the LNs at the peripheral subcapsular sinus and also via the high endothelial venules, which support lymphocyte entry from the blood [1, 2]. The LN is a dynamic organ capable of undergoing dramatic remodelling, in terms of both architecture and function, in response to pathological conditions such as inflammation or cancer [3]. Many solid cancers spread through the lymphatic system to distant organs, with the LNs typically serving as a first site of seeding outside primary tumour [4–6]. For these tumours, the presence and extent of LN metastasis are markers of aggressive phenotype, often having an inverse linear relationship with prognosis [7–9]. In breast carcinoma patients, metastasis to LN is an important factor for staging the tumour and routine assessment for invasive breast carcinoma patients includes histopathological assessment of the presence of metastasis, the number of involved LNs and the presence or absence of extra-nodal extension [10].

Although the LN is a functional organ for tumour–immune system interaction and may be a read-out for systemic immune responses, studies of the molecular characteristics of LNs have centred around mutational alterations and structural genome rearrangements, whereas transcriptional research has been limited in both human and pre-clinical models [11]. Most studies have aimed to identify molecular signatures associated with good and bad prognosis in primary breast tumours, and gene sets consistently predicting the development of LN metastasis have yet to be determined [12–17], while the genomes of relapsed or secondary breast cancers have revealed that metastases and primary tumours are clonally related, share several driver mutations and often acquire additional novel variants that are not present in the primary lesion [18].

In the metastatic LN, a multitude of factors play important roles in tilting the balance between pro-metastatic immunosuppression and anti-tumoural immune response [19–21]. Given the significant implication of LN metastasis for systemic cancer burden, surprisingly little emphasis has been given to elucidate the underlying molecular signals and cellular alterations of the evolving LN microenvironment between the uninvolved (cancer-free) and the involved (metastatic) LNs in breast cancer patients. Some of these changes include lymphangiogenesis and increased lymph flow [22], recruitment and expansion of immunosuppressive cells (including myeloid-derived suppressor cells and regulatory T cells) [23], upregulation of chemokines and cytokines, blood vessel remodelling [24, 25] and a lower percentage of effector T cells [26]. We recently comprehensively histologically characterised diverse immune and stromal features in primary tumours and their associated involved and uninvolved axillary LNs in a cohort of 309 invasive breast cancer patients (143/309 LN positive) [27] and observed that architectural alterations of the uninvolved LN are significant predictors for distant metastases. A similar finding of prognostic information from examination of the LN architecture was observed in melanoma [28]. In preclinical mouse models, the involvement of innate lymphoid RORγt^+^ ILC3 cells, fibroblast reticular cells and cancer-associated fibroblasts in the induction of an immunosuppressive and pro-metastatic microenvironment in tumour-draining LNs was reported [29–31], while uninvolved regional LNs in rats with prostate tumours displayed varying degrees of genetic changes depending on prostate tumour groups and their metastatic capacity [32].

With regards to emerging immunotherapy approaches, the LN microenvironment and the nature of the immune response have been identified as potent indicators of response to therapeutic interventions [33, 34]. With the central position of the LN as an immune organ and as a gateway for further dissemination of tumour cells to other metastatic sites, we conducted a comprehensive review of existing gene expression-based research performed on LNs in human breast cancers. We categorised these gene expression studies along the evolving microenvironment of axillary metastases. By starting with early colonisation to the replacement of the entire LN with metastasis, these expression patterns capture information on the molecular mechanisms and changes in immune composition that allow the exploration of LNs as a pro-metastatic niche. Since patients with locoregional breast cancer typically have a high risk of developing distant metastasis and thus poor overall survival, it is particularly important to establish whether transcriptomic patterns indicative of metastasis might translate into new therapeutic strategies, including the successful implementation of immunotherapy.

## Materials and methods

### Literature search and data collection

**Fig. 1:**
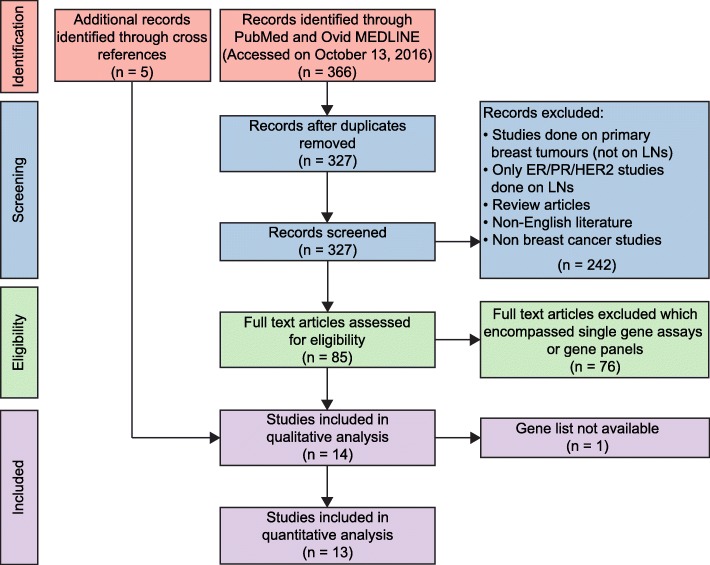
Systematic review flowchart in accordance with the PRISMA statement [35] for the gene expression studies performed on LNs in human breast cancer patients. A total of 14 studies were included after the procedure of searching, screening and excluding from the English literature database. Thirteen of these studies were subjected to quantitative analysis

A review of the English literature was performed, focusing on gene expression data derived from human LN tissue and the primary lesion in breast cancer patients (if matched LN tissue was interrogated), using the combination of the following keywords: “breast cancer”, “metastasis”, “lymph nodes” and “gene expression” in “all fields” in PUBMED and Ovid MEDLINE ® (accessed on 13th October 2017 and revised on 5th June 2018). All abstracts were manually screened and their methodologies were reviewed. Papers were selected if genome-wide (i.e. microarray or RNA-sequencing based) gene expression analyses of LNs of breast cancer patients were performed (*n* = 14). Studies of primary breast tumours and distant metastatic sites which reported only the LN status of the patients were excluded (see consort diagram in Fig. 1). The review was conducted according to the preferred reporting items for systematic reviews and meta-analyses (PRISMA) statement [35].

### Data analysis

Of a total 366 papers screened, 14 studies were included in the review: Calvo et al. [36], Feng et al. [37], Hao et al. [38], Lähdesmäki et al. [39], Weigelt et al. [40], Ellsworth et al. [41], Vecchi et al. [42], Suzuki et al. [43], Mathe et al. [44], Zuckerman et al. [45], Blackburn et al. [46], Valente et al. [47], Rizwan et al. [48] (all of which performed microarray-based gene expression analyses); and Liang et al. [49], which used 18–27 million paired-end riboZero RNA-sequencing. Genes with differential expression between the respective scenarios were obtained directly from the publications; no cut-offs were applied (Table 1). Using the biomaRt R package [50, 51], either gene names or microarray features were converted to ENSEMBL ID (ENSEMBL GRCh37.p13) [52] (Additional files 1, 2 and 3: Tables S1–S3). If microarray features could not be mapped, assuming that their sequences are retired (i.e are not present in any current sequence database), they were excluded from further studies. Once an ENSEMBL ID list was created, HGNC symbols, genomic location and their common gene ontology terms were recorded. From these ENSEMBL gene lists, pathway analyses were conducted on de-regulated genes using the WebGestalt tool [53] (Additional file 4: Table S4). The overrepresentation analysis (ORA) was applied based on the *Homo sapiens* Gene Ontology (GO) biological processes database. The whole genome was used as a reference; all GO terms < 0.05 FDR were extracted. To remove redundant GO terms, the Revigo tool with parameter “small” was used [54]. The resultant GO terms and differentially expressed genes were compared between the groups. To capture genes representative for specific immune cell populations, the gene lists compounded from the studies were cross-referenced with published immune metagenes [55].

**Table 1:** Genome-wide expression studies of LNs of breast carcinoma patients

Clinical question	Study	Breast carcinoma	Sample cohort	Results
Scenario 1 Involved lymph node (ILN) versus primary tumours (PT)	Calvo et al. [36], 2013	IDC	18 PT vs matching ILN	Infrequent loss of luminal differentiation in metastatic LN
	Feng et al. [37], 2007	IDC	26 PT vs matching ILN	79 DEG
	Hao et al. & Lähdesmäki et al. [38, 39], 2004	Invasive BC	9 PT vs matching ILN	280 DEG
	Weigelt et al [40], 2005	Invasive BC	15 PT vs matching ILN	No classifier or single gene could discriminate
	Ellsworth et al. [41], 2009	Invasive BC	20 PT vs matching ILN	51 DEG
	Vecchi et al. [42], 2008	Invasive BC	26 PT vs matching ILN	270 DEG
	Suzuki et al. [43], 2007	Invasive BC	10 PT vs matching ILN	84 DEG
Scenario 2 Involved LN versus normal adjacent breast tissue (NAT)	Mathe et al. [44], 2015	TNBC	15 ILN vs 17 NAT	83 genes were significantly associated with LN metastasis
Scenario 3 Uninvolved LN in LN-positive versus LN-negative patients	Zuckerman et al. [45], 2013	Invasive BC	11 PT, 30 LN, 21 PB	116/219 DEG (SLN/NSLN, respectively)
	Blackburn et al. [46], 2017	Invasive BC	24 LN from NP vs 40 LN from NN	No genes were differentially expressed with stringent FDR
Scenario 4 Uninvolved residual portion of involved LN versus uninvolved LN	Valente et al. [47], 2014	Invasive BC	20 matched pairs of involved and uninvolved LN	22 DEG
	Zuckerman et al. [45], 2013	Invasive BC	11 PT, 30 LN, 21 PB	103 DEG
Scenario 5 Involved LN versus uninvolved LN	Rizwan et al. [48], 2015	Invasive BC	16 involved vs 3 uninvolved LN	13 DEG
Scenario 6 Positive sentinel LNs in patients with additional, non-sentinel, positive LNs to patients with additional, non-sentinel, negative LNs	Liang et al. [49], 2015	Invasive BC	3 NSLN+ SLN vs 3 NSLN− SLN	160 DEG

*BC* breast carcinoma, *DEG* differentially expressed genes, *IDC* invasive ductal carcinoma (no special type), *ILN* involved LN, *LN* lymph node, *NAT* normal adjacent breast tissue, *NN* node-negative patients, *NP* node-positive patients, *NSLN* non-sentinel lymph node, *PT* primary tumour, *SLN* sentinel lymph node

## Results and discussion

### Overview of expression profiling studies on LNs in breast cancer

**Fig. 2:**
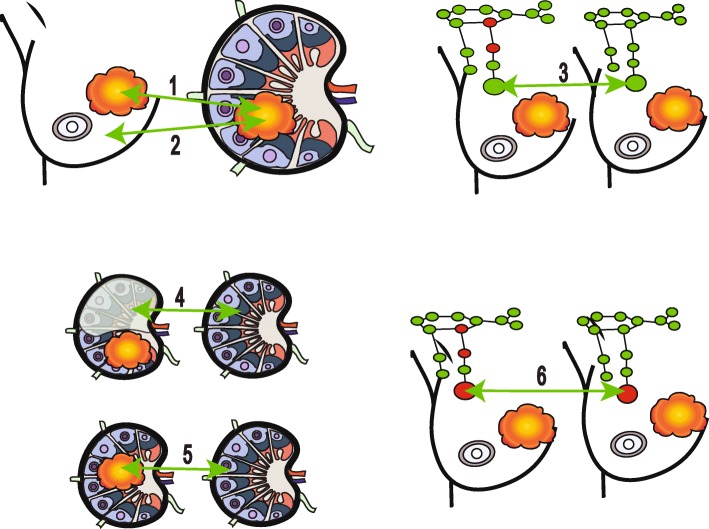
Different scenarios for studying lymph nodes, breast cancers and normal tissue. Six scenarios depict different comparisons (indicated by *green arrows*): scenario 1, involved lymph node versus primary tumour (number of studies = 8); scenario 2, involved lymph node versus normal breast tissue (number of studies = 1); scenario 3, uninvolved LNs in LN-positive patients versus uninvolved LNs in LN-negative patients (number of studies = 2); scenario 4, uninvolved residual portion of involved LN versus patient-matched uninvolved LN (number of studies = 2); scenario 5, involved LN versus patient-matched uninvolved LNs (number of studies = 1); scenario 6, involved sentinel LNs in patients with additional, non-sentinel, positive LNs versus involved sentinel LNs in patients with additional, non-sentinel, negative LNs (number of studies = 1). Tumours are shown in *orange* and *red* and *green* denote involved and uninvolved LNs, respectively. In scenario 4, the shaded portion represents the uninvolved residual portion of an involved LN

A total of 14 genome-wide transcriptomic studies on LN samples were selected to decipher the molecular features of the evolving LN microenvironment as a locoregional metastatic site [36–49]. Each article published lists of genes specifically transcriptionally activated or repressed in LNs, ranging from cancer-free to metastatic settings. The cohorts were of mixed-receptor (Estrogen (ER), Progesterone (PR) and Human epidermal growth factor receptor (HER2)) invasive breast carcinomas, including two studies of invasive carcinomas of ductal/no special type only and one exclusively examining triple negative breast carcinomas (TNBC). To paint a chronological picture of the changing microenvironment of the evolving metastatic LN, the studies were grouped into six “scenarios”, described below in detail (Table 1, Fig. 2).

#### Scenario 1: Comparison between involved LN and primary breast carcinoma, the drivers of metastasis

With the common aims of searching for drivers of metastatic progression, developing metastatic signatures predictive of distant metastasis [37, 42] and identifying molecular targets for metastasis-specific therapy or markers of resistance, eight of 14 studies captured transcriptional alterations between involved LNs and their patient-matched primary carcinoma. Expression patterns and gene regulatory pathways potentially driving metastatic dissemination were determined, while the point of acquiring metastatic efficiency in a primary tumour’s timeline was intended to be revealed. These studies focussed on the cancerous tissue itself rather than the LN microenvironment; thus, the material selected for analyses had at least 70% tumour tissue, or laser microdissection was performed.

Although high transcriptomic similarity between primary carcinoma and its corresponding LN metastasis was consistently observed [36, 39–41, 43], genes exclusively expressed in either of these two cancerous tissues was reported. Taking into consideration the diversity of the clinical characteristics of these cohorts, we asked whether any commonalities among activated or repressed genes could be established, potentially pointing collectively to deregulated biological themes. Among the eight studies, a total of 88 genes were found to be differentially expressed between the involved LN and the primary tumour in at least two studies, while the downregulation of 21 genes associated with cell–extracellular matrix (ECM) interaction, ECM remodelling, epithelial–mesenchymal transition (EMT) and loss of basement membrane function [56, 57] was common to three studies (Additional files 1 and 2: Tables S1 and S2).

Downregulation of EMT-associated genes in the involved LN might suggest that, as the metastasis becomes established, reversal of EMT and restoration to epithelial phenotype are essential for the successful colonisation [47]. Stromal cells play a significant part in this process, particularly matrix metalloproteinases MMP2 and MMP7, as these proteins are associated with the breakdown of the ECM, as well as innate immune response [58]. CD10, a membrane metalloendopeptidase, is present at various stages of B-cell maturation and of particular importance in LNs, where it is strongly expressed by germinal centre B cells, the most highly proliferative lymphocyte subset in LNs [59]. CD10 was less abundant in involved LNs compared to the primary lesions in three studies [36, 41, 42], potentially pointing to a lack of differentiation potential of B cells.

Three genes, namely those encoding collagenase 11A1 (*COL11A1*), Asporin (*ASPN*) and Periostin (*POSTN*), were reported in four studies as having lower abundance in involved LNs compared to primary tumour tissue [37, 38, 41–43]. All three genes function in remodelling ECM and ECM-associated protein degradation of the basement membrane. ECM remodelling is a well-established mechanistic prerequisite for dissemination of the primary cancer and genes involved in ECM are frequently part of metastatic gene sets in several other solid tumours [60]. *COL11A1* promotes cell proliferation, migration and tumourigenesis of many human malignancies [61]. This gene is currently being investigated as a diagnostic marker for non-small cell lung carcinoma (NSCLC) and, by targeting *COL11A1*, chemoresistance might be overruled [62]. The stromal expression of *ASPN* and *POSTN* has been shown to be associated with aggressive tumour phenotypes and poor prognosis in prostate and colorectal cancers, respectively [63, 64]. Whether their lack of expression in involved LNs provides additive risk information for disease progression warrants further investigation.

Complement component 7 (C7), a protein involved in the innate immune system, and part of the membrane attack complex that mediates lysis of pathogens, was the only gene of higher abundance in involved LNs reported in four studies [37, 41–43]. Since C7 may be related to processing and responding to different tumour neo-antigens present in involved LNs, its presence might reflect attempts of the involved LN to counterattack the metastatic colonisation.

**Fig. 3:**
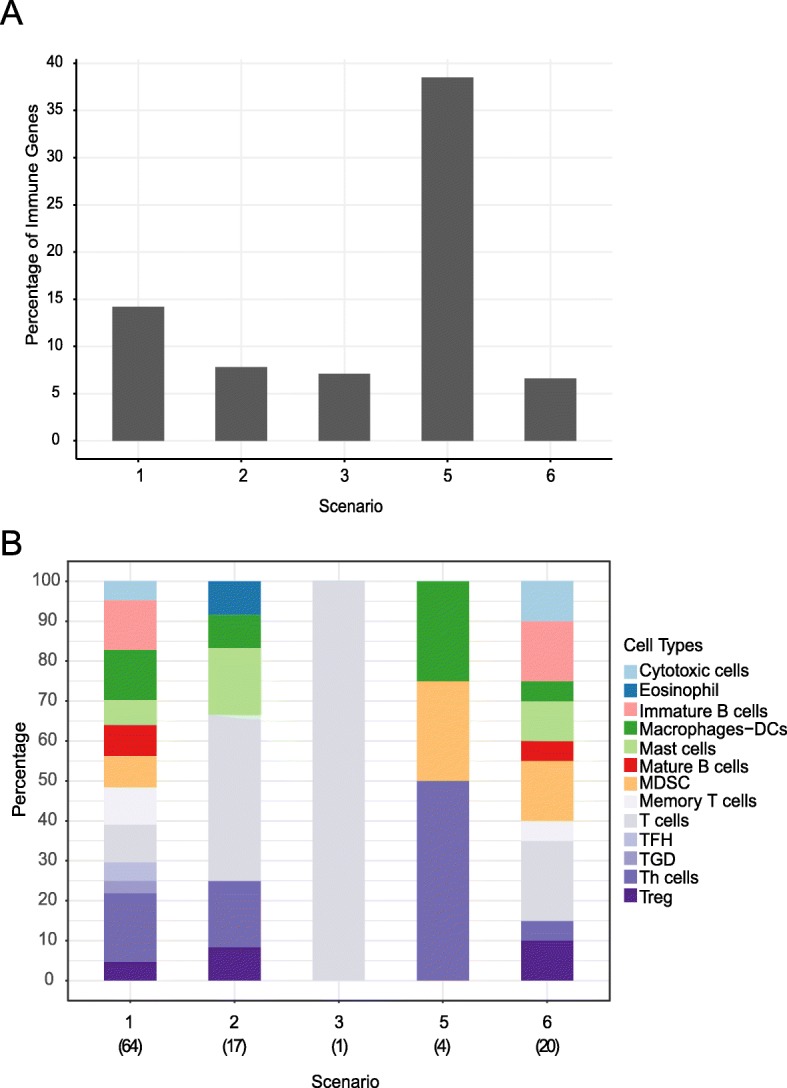
Immune cell composition in different scenarios. **a** The percentage of genes representing specific immune cell populations in each of the scenarios. **b** The proportion of different immune cell populations among all the immune-related genes in each scenario. (Scenario 4 was omitted as the reported 103 differentially expressed genes could not be retrieved from the original study)

Besides the malignant epithelial component, the transcriptional profiles of involved LNs almost always still harbour significant signals of immune and stroma cells. Among all eight studies, a total of 64 immune cell-related genes were identified (Fig. 3a, b, Additional file 5: Table S5), including those associated with the upregulation of chemokines, ligands and receptors, cytotoxic CD8+ T cells, both immature and activated B cells, T-cell receptor (TCR) activation, MHC class II, Th1 and Th2 cells in involved LNs. Conversely, genes downregulated in involved LNs were associated with dendritic cells (DCs), mast cells and monocytes. DCs are antigen-presenting cells that enter the LNs via the afferent lymphatics and that prime the effector T cells to initiate adaptive immune responses. Germinal centre responses are dependent on T cells activated by DCs. A depletion of DCs could represent a major immune escape mechanism in cancers [65] due to lymphangiogenic responses in the metastatic node [66]. A previous study found that not only the number but also the spatial clustering of dendritic cells in tumour-draining LNs affects clinical outcome of breast and other cancer patients [67]. In melanoma, for example, decreased numbers of plasmacytoid DCs (pDC) in peripheral blood had independent negative prognostic value mainly linked to stage IV disease and with associated gradual decline in pDC levels just before relapse [68]. Indeed, a multitude of factors, including number, spatial organisation, migration and maturation status of DCs, play a pivotal role in determining the anti-tumour immune response/pro-tumour immunosuppression balance [69, 70]. Conventional dendritic cell type 1 (cDC1) is the key player in stimulating CD8+ T cells and inducing antitumor T-cell responses [71], and various subtypes of blood derived LN-resident DCs (pDC, Clec9A+ DCs, BDCA+ DCs) can induce both Th1 and Th2 cytokines [72]. Thus, a dynamic interplay with the modulation of humoral and cellular immune responses, histologically corroborated by the reactive nodal changes with follicular, paracortical and sinusoidal hyperplasia, is present in these involved LNs [27].

Overall, our unifying analyses repeatedly demonstrated a consistent plasticity in ECM and immune cells in metastatic LN tissue, despite the underlying molecular similarities between the primary carcinomas and patient-matched involved LNs. Cancer genomes reflect clonal persistence and clonal extinction during cancer evolution [18]. A recent comprehensive single cell analysis of chemoresistant TNBC supported an evolutionary model in which an adaptive selection in the cancer genome is paralleled by an acquired transcriptional program, including ECM degradation and EMT [73]. Given the remarkable molecular similarities between primary lesions and involved LNs, the metastatic genetic programme may be activated at an early stage during breast cancer development [15, 74], some cancerous cells may acquire their metastatic proficiency late due to clonal evolution [75], and as a sum are continually reshaping the metastatic molecular expression profile [43]. In parallel, as the metastatic potential of these cells evolves and increases over time, and the local microenvironment, through the interaction with endothelial, stromal and immune cells, carries significant determinants for successful colonisation in the LN.

#### Scenario 2: Comparison with normal breast tissue, pinpointing the changes in metastasis

To decipher the remarkable similarities between a breast primary tumour and its LN metastasis, Mathe and colleagues [44] made multiple comparisons between normal breast tissue, LN-positive primary tumours, LN-negative primary tumours and LN metastases. Their hypothesis for identifying genes crucial for metastatic spread relied on: (i) genes differentially expressed between primary tumour versus normal, tumour-adjacent breast tissue (NAT) in a LN-positive patient, followed by (ii) genes expressed in involved LN compared to normal breast tissue, and then (iii) selecting only those genes which were absent in primary tumours versus normal breast tissue in LN-negative patients. Through this step-wise approach, 14 genes were found commonly as downregulated in involved LNs (*APOD*, *MME*, *OMD*, *F2RL2*, *DCN*, *PTN*, *SFRP2*, *FMO1*, *OGN*, *SRPX*, *SPARCL1*, *MMP16*, *LRRC1*, *HMCN1*; Additional file 3: Table S3). *SPRX*, *SPARCL1*, *MMP16* and *HMCN1* are again involved in cell adhesion, ECM breakdown and organisation. *DCN* influences regulatory T cell (Treg)-mediated immunosuppression, while *CD10*, as noted above, is essential for highly proliferative and pro-apoptotic germinal centre B cells [59, 76].

Performing an overrepresentation analysis using the GO database [53], pathways frequently deregulated in involved LNs in both scenarios 1 and 2 included ossification, cell adhesion, ECM organisation, cell proliferation, cell motility, apoptotic process and development of vasculature. Remodelling of the ECM and vascular proliferation are corroborated by the histological alterations in stromal architecture seen in LNs when metastasis manifests itself (Additional file 4: Table S4) and have previously been linked to metastasis in multiple solid tumours [77].

In parallel, a delicate balance between helper and regulatory T cells seems to create a pro-metastatic immunosuppressive niche in the LN, as identified by seven downregulated (*EGR1*, *RBMS3*, *CD34*, *IGF1*, *MEIS2*, *CMA1*, *DLC1*) and five upregulated (*MAD2L1*, *STAT1*, *KIF11*, *ANLN*, *DLGAP5*) genes associated with specific immune cell populations, especially T-cell function including helper (*RBMS3*, *DLC1*) activated (*MAD2L1*, *KIF11*, *ANLN*, *DLGAP5*) and regulatory T cells. Different subsets of helper T cells, including Th17 and the heterogeneity of Tregs, are critical for cancer progression and metastasis [78, 79], again emphasising that the balance between different subsets of helper and regulatory T cells is a crucial factor in successful colonisation.

##### Gene expression patterns across different phenotypical LN groups

By exclusively studying the involved LNs, key questions of “when” does the LN microenvironment develop signals to potentially attract cancer cells and when, why and how these cancer cells can home in in such an immune cell-dominant environment are omitted. LNs at different stages of colonisation provide the opportunity to obtain insight into the underlying biology of the evolving pre-metastatic setting. The following four scenarios adopted the diverse approaches across nodes of different status (Fig. 2):

Scenario 3: By comparing uninvolved LNs in LN-positive and LN-negative breast cancer patients**,** the premetastatic niche and early genetic aberrations were interrogated for changes in immune response, vasculature and cellular proliferation, which are potentially measureable even before detectable metastasis has occurred. Here, molecular changes specific for a node-to-node manner and alterations systemically affecting the regional nodes can be determined [45–47].

Scenario 4: Comparison between the uninvolved, residual portion of a LN bearing a metastatic carcinoma with patient-matched negative nodes allowed identification of late-stage alterations in the secondary microenvironment, which may indirectly support metastatic growth [45, 47].

Scenario 5: By comparing involved LNs with uninvolved LNs, alterations of immune and stromal cells within similar secondary microenvironments are captured [48].

Scenario 6: By relating positive sentinel LNs in patients with additional, non-sentinel, positive LNs to patients with additional, non-sentinel, negative LNs, gene patterns conferring increased risk of developing metastasis in other LNs might be delineated [49].

#### Scenario 3: The uninvolved LN, the first step towards metastasis

The first step in the colonisation of the LN by tumour cells is potentially the preparation of the LN microenvironment, even before the tumour cells arrive. Blackburn et al. [46] and Valente et al. [47] investigated the transcriptomic profiles of uninvolved LNs in LN-positive and LN-negative patients to identify early preparatory changes in the LN microenvironment. Both studies did not observe significant differences in gene expression patterns between the uninvolved LNs of LN-positive versus uninvolved LNs of LN-negative breast cancer patients [46, 47], leading the authors conclude that (a) the physical presence of metastatic tumour cells may be crucial to elicit a pro-metastatic niche in the LNs and (b) these pro-metastatic changes occur in a LN-to-LN manner and are not reflected systematically in uninvolved LNs in an otherwise LN-positive patient.

Studying the early metastatic changes, Zuckerman et al. [45] followed a different approach by purifying immune cells from uninvolved sentinel and non-sentinel LNs. In uninvolved LNs (of entirely LN-negative patients), gene patterns were associated with immune cell regulation and signalling pathways such as antigen presentation (*HLA-DQA*, *HLA-A*, *HLA-DRB3*), lymphocyte activation (*HLA-DOA*, *IL23A*, *IL4*, *PLCG2*, *TICAM1*), cytokine–cytokine receptor interaction (*IL12RB2*, *IL4*, *CCR8*, *TNFRSF21*, *IL23A*, *IL3RA*) and pro-inflammatory TREM1 and IL-17 signalling [80, 81], indicating an effective antigen-processing and anti-tumour response. TREM1 signalling activates monocyte-macrophage and neutrophil-mediated immune responses. The IL-17 pathway stimulates Th17 cells to respond to a variety of foreign antigens and is involved in autoimmune diseases [82]. Activation of such pro-inflammatory immune pathways in a LN-negative patient’s LNs may facilitate an effective tumour response that prevents successful further spreading and colonisation of metastatic cells. In this context, breast cancer cells have been shown to hinder the functioning of dendritic cells and other antigen-processing cells [83]. In contrast, the uninvolved LNs of LN-positive patients had higher levels of genes involved in relaxin signalling, which attracts mononuclear cells to create an immunosuppressive environment [84]. The lack of effective immune responses, including antigen presentation, together with tumour promoting factors may all synergise to establish the necessary immunosuppressive pre-metastatic niche in the uninvolved LN of LN-positive patients. These molecular alterations may cause various architectural changes, including changes in size and location of germinal centres in uninvolved LNs of LN-positive breast cancer patients, as we have observed previously [27].

#### Scenario 4: Residual portion of an involved LN, a surviving immune microenvironment

**Fig. 4:**
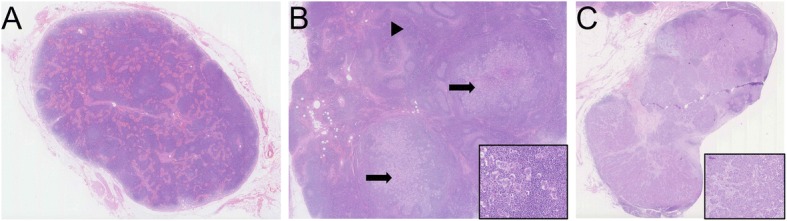
Chronological steps of lymph node metastasis (H&E stain). **a** An uninvolved axillary LN with no evidence of tumour cells (0.7×). **b** Partial colonisation of a LN with significant amount of residual uninvolved LN tissue (*black arrowhead*) and two nodules of metastasis (*black arrows*) are depicted (0.5×). Inset shows tumour cells mixed with background immune cells (20×). **c** A lymph node with near total replacement of normal lymph nodal tissue (1×). The inset displays a higher power magnification of tumour cells (10×). All images were captured by Nanozoomer and viewed in NDP.view2 software (Hamamatsu)

A reflection of the vanishing immune cell microenvironment from the uninvolved to the involved LN is provided by assessment of the residual portion of a LN where some colonisation by tumour cells has started (Figs. 2 and 4). The uninvolved, ‘normal’ residual portion of an otherwise involved LN offers a unique snapshot of direct interaction between LN stromal and immune cells with tumour cells. To study the gene expression exclusively from this area of the LN, Valente et al. [47] confirmed the absence of tumour cells with AE1/AE3 immunohistochemical staining and laser microdissected the cancer-free tissue for RNA extraction. Similarly, Zuckerman and colleagues carefully selected, with flow cytometry-based sorting, only immune cells from residual LN materials [45]. Most genes downregulated in the residual parts of involved LNs, when compared to completely uninvolved LNs, were involved in regulation of immune response (*HPGDS*, *STAB2*, *CLEC4M*, *PROS1*, *TFPI*), advocating a pro-metastatic immunosuppressive microenvironment. *STAB2*, a scavenger receptor, is known to regulate leukocyte trafficking in LNs through lymphatic endothelial cells [85], theoretically maintaining defence and tissue homeostasis, and in parallel spreading neoplastic cells. Similarly, in uninvolved LNs of otherwise LN-positive patients, pathways downregulated in the residual portion of positive LNs were pro-inflammatory immune-related pathways like TREM1 signalling (*NOD2*, *TLR5*), whilst the upregulated pathways were associated with cell cycle (*RAD51*, *KIF23*, *PLK4*), DNA repair (*RFC2*, *BRIP1*) and tumour-promoting angiopoietin signalling (*RASA1*, *BRIP1*). In residual LN tissue (from nodes with metastatic tumour) compared to uninvolved LNs, B-cell-related genes (*AICDA*, *IGKC*, *IGKV1-5*, *IGKV3-20*), many of them specifically expressed in germinal centres, were highly active. B cells and ectopic germinal centres have previously been linked to chronic inflammation and tumour promotion [86, 87] and may represent prognostic indicators for developing distant metastases (Figs. 2 and 4) [27, 28]. The upregulation of cell cycle and DNA repair pathway genes can further be linked to germinal centres, as these are zones of high proliferation. One might hypothesise that, in uninvolved LNs of LN-positive patients and in the residual ‘normal’ part of an involved LN, the upregulation of germinal centre B cell genes, in parallel to the dampening of antigen presentation and T-cell priming, results in an altered tumour-promoting response, primarily mediated by B cells. Defective immune regulation in which B-cell proliferation or humoral response is activated, in spite of the dampening of the antigen presentation and leukocyte activation, through some alternative pathways could create a pro-metastatic environment. Furthermore, the abundance of kappa light chain genes as overexpressed in residual LN tissue point to an alternative B-cell activation pathway biased towards B cells expressing kappa light chains and of oligoclonal nature. In the presence of B-cell proliferation, it is essential to study markers such as PD-1, a negative regulator of B-cell differentiation and expressed by the majority of T cells in germinal centres. B cells can both positively and negatively regulate T-cell-mediated antitumor immune responses; however, their function in generating a specific pre-metastatic niche has yet to be established [66].

#### Scenario 5: From an uninvolved to an involved LN status

To study the penultimate step in the evolving LN microenvironment one can look at the extreme endpoints, i.e. to capture transcriptional changes in the involved LN as a whole and compare with the uninvolved LN. Rizwan and colleagues mainly focussed on change in collagen density in LNs in a murine metastatic breast cancer model, and examined expression patterns derived from publicly available microarray-based data (GSE4408), in which 16 involved and three uninvolved human LNs from breast cancer patients were compared [48]. Ten of the 14 genes transcriptionally activated in involved LNs were fibronectin (*FN1*), three collagen genes (*COL1A2*, *COL1A1*, *COL3A1*) and six integrin family members (*ITGB5*, *ITGA2*, *ITGA9*, *ITGB7*, *ITGA2B*, *ITGA4*). All are key players in cell adhesion, cell–ECM interaction and ECM modulation (Additional files 3 and 4: Tables S3 and S4). Involved LNs displayed increased collagen I and basement membrane density in this murine metastatic breast cancer model. Increased collagen can promote tumour spread, not only by augmenting cell motility and regulating tumour promoting cell–ECM interactions, but also by altering immune responses, including switching the phenotypes of macrophages to a tumour-promoting M2 type [88] as well as a reduction of B-cell follicles [48].

#### Scenario 6: The final step—can involved LNs send signals to other uninvolved LNs to promote tumour dissemination?

The number of involved LNs in breast cancer is associated with the risk of developing distant metastasis [7]. The prediction of the extent and number of involved non-sentinel LNs by assessing the sentinel LN(s) would potentially have practical clinical importance, as axillary LN dissection in a group of sentinel LN-positive patients could be avoided [89, 90]. The study by Liang and colleagues, although performed on only six patients, addressed the question of whether completely replaced LNs, especially the sentinel LNs, could send ‘signals’ to uninvolved LNs in preparation to disseminate the tumour cells [50]. By comparing involved sentinel LNs in patients with additional metastasis in non-sentinel LNs to those with otherwise negative axillary (non-sentinel) LNs, tumour-promoting pathways were represented in the non-sentinel LN-positive group, indicated by the expression of kallikrein subfamily members (*KLK10*, *KLK11*, *KLK12*, *KLK13*), proteolysis and steroid receptor signalling. In contrast, genes involved in plasma membrane and B cell receptor signalling, including *CD22*, *CD72*, *Igα*, *Igβ*, *CD19* and *CD21*, were depleted in parallel with *SYK*, *LYN*, *BTK* and *PTPN6*. In the group of patients with additional positive LNs, specific gene fusions were noted, especially involving *IGLL5*, a surrogate light chain involved in B-cell development [91]. Using immune metagenes denoting specific immune cell populations [55], an overlap between immature and activated B cells (*FCRLA*, *FAM129C*, *CD22*, *PAX5*), helper T cells (*SIGLEC10*), MDSCs (*CEACAM8*, *FCER2*), mast cells (*CLC*, *SIGLEC14*) and regulatory T cells (*CD72*, *IL9R*) (Additional file 5: Table S5) was observed. Taken together, a recurrent theme for further tumour cell spreading emerges in these gene expression patterns, pointing strongly to a key role of B cells and germinal centres in LNs. Accumulating evidence supports a role for B cells in breast cancer immunology [92], and therapeutic approaches targeting B cells may before long demonstrate their relevance. Already in 2015, Sagiv-Barif and colleagues reported substantial enhanced anti-tumour responses in the 4T1 TNBC mouse model when treated with a combination of anti-PDL-1 with ibrutinib, an inhibitor of Bruton’s tyrosin kinase (*BTK*) [93], an essential kinase for B cell maturation, signalling, and graft-versus-host disease [94]. Clinical trials (e.g. ClinicalTrials.gov NCT02403271) are currently evaluating B-cell depletion or *BTK* inhibition along with checkpoint inhibition and will soon expose whether such combination therapies enhance anti-tumour immunity and potentially even reduce checkpoint inhibitor-associated treatment-related toxicities in breast cancers [95].

### LN, a read-out for the systemic immune response?

Being an early site of tumour dissemination, the LN hosts a variety of tumour–immune system interactions. The ultimate question remains whether certain patterns in LNs of breast cancer patients’ mirror changes in the systemic immune response to the tumour in the organism. Valente et al. [46] and Blackburn et al. [47] argued that the physical presence of cancer cells in the LN is crucial for pre-metastatic niche development and that the changes are therefore not systemic. However, recent research, such as the presence of similar immune gene sets in the uninvolved LNs in LN-positive patients and the residual tissue of involved LNs [45], in addition to peripheral blood and to some extent in the immune compartment of the primary tumour [45], identified changes most likely indicative of a systemic effect in LN-positive patients. In keeping with this hypothesis, work on systemic immune responses to effective immunotherapies in preclinical murine breast cancer models has proven experimentally that changes in the immune composition persist in primary tumours, regional LNs, peripheral blood, bone marrow and other lymphoid organs [34].

### Limitations

Despite the scarcity of expression data from LN tissue of breast cancer patients, together these data expose snapshots of the steps of the molecular transitions that occur, starting from the uninvolved LN in LN-positive patients, to uninvolved residual tissue of involved LNs, to fully involved LNs, and finally the pro-disseminating signals in involved LNs. Ideally, all these comparisons should be examined within an individual patient’s samples to exclude patient-to-patient heterogeneity. Genome-wide studies of whole LN samples mask effects in this highly spatially organised immune organ. Using sophisticated imaging technologies or single cell -omics analyses to capture the earliest stages of LN metastasis, i.e. when tumour cells enter through the afferent lymphatic vessels and colonise in the subcapsular sinus [96], would provide valuable biological and potentially clinically relevant information.

## Conclusion

The prognostic relevance of changes in uninvolved LNs is tantalising as it highlights the need to study the interconnected roles of immune, stromal and endothelial cells within this small immune organ as well as the whole immune system [27, 28]. With the recent findings of the systemic orchestration of immune cells with effective immunotherapy [34], examination of local plus systemic tumour–immune cell interactions might hold the key for successful immunotherapeutic strategies. Although some patterns are evident from close scrutiny of existing literature, the ‘premetastatic’ LN represents an unmet knowledge gap; comprehensive cellular and molecular studies focusing on changes in different immune cell compartments at different time-points during the development of metastasis are needed to unlock this complicated biological process, from both a mechanistic and therapeutic point of view.

## Additional files

Additional file 1: **Table S1.** Gene list compiled from all the studies included in scenario 1 (involved LN versus primary breast tumour). (PDF 75 kb)

Additional file 2: **Table S2.** Genes found to be differentially expressed in multiple studies included in scenario 1 (involved LN versus primary breast tumour). (PDF 18 kb)

Additional file 3: **Table S3.** Fully compiled gene list across all scenarios. (PDF 75 kb)

Additional file 4: **Table S4.** Pathway-based analysis of the differentially expressed genes across all the scenarios. (PDF 33 kb)

Additional file 5: **Table S5.** Differentially expressed genes representing specific immune cell populations across all the scenarios. (PDF 21 kb)
