# Supplementary material for: Molecular patterns of cancer colonisation in lymph nodes of breast cancer patients
Source: Breast Cancer Res. 2018 Nov 20;20:143. doi: 10.1186/s13058-018-1070-3 (PMC6247766; doi:10.1186/s13058-018-1070-3)
Supplement: Supplementary file 1 — Table S1. Gene list compiled from all the studies included in scenario 1 (involved LN versus primary breast tumour). (PDF 75 kb) [file 13058_2018_1070_MOESM1_ESM.pdf]

Gene Name	Gene ID	Change	Study	Ensembl gene ID
APOD	NM_001647	DOWN	Calvo (37)	ENSG00000189058
KRT5	NM_000424	DOWN	Calvo (37)	ENSG00000186081
C11orf43	X07868	DOWN	Calvo (37)	ENSG00000167244
TRIM29	NM_012101	DOWN	Calvo (37)	ENSG00000137699
IL8	NM_000584	DOWN	Calvo (37)	ENSG00000169429
FBLN1	Z95331	DOWN	Calvo (37)	ENSG00000077942
FBLN1	NM_006486	DOWN	Calvo (37)	ENSG00000077942
ANXA8	NM_001630	DOWN	Calvo (37)	ENSG00000265190
F13A1	NM_000129	DOWN	Calvo (37)	ENSG00000124491
MME	NM_007287	DOWN	Calvo (37)	ENSG00000196549
DIO2	U53506	DOWN	Calvo (37)	ENSG00000211448
HEPH	NM_014799	DOWN	Calvo (37)	ENSG00000089472
HPGD	NM_000860	DOWN	Calvo (37)	ENSG00000164120
HPGD	NM_000860	DOWN	Calvo (37)	ENSG00000164120
GULP1	NM_016315	DOWN	Calvo (37)	ENSG00000144366
S100A2	NM_005978	DOWN	Calvo (37)	ENSG00000196754
DST	NM_001723	DOWN	Calvo (37)	ENSG00000151914
GAS1	NM_002048	DOWN	Calvo (37)	ENSG00000180447
MMP1	NM_002421	DOWN	Calvo (37)	ENSG00000196611
VSIG4	NM_007268	DOWN	Calvo (37)	ENSG00000155659
GABRP	NM_014211	DOWN	Calvo (37)	ENSG00000094755
KRT17	NM_000422	DOWN	Calvo (37)	ENSG00000128422
CPA3	NM_001870	DOWN	Calvo (37)	ENSG00000163751
MMP10	NM_002425	DOWN	Calvo (37)	ENSG00000166670
OMD	NM_005014	DOWN	Calvo (37)	ENSG00000127083
DSPG3	NM_004950	DOWN	Calvo (37)	ENSG00000083782
F2RL2	NM_004101	DOWN	Calvo (37)	ENSG00000164220
KCND2	NM_012281	DOWN	Calvo (37)	ENSG00000184408
TNFSF4	NM_003326	DOWN	Calvo (37)	ENSG00000117586
DCN	BC005322	DOWN	Calvo (37)	ENSG00000011465
CHI3L1	M80927	DOWN	Calvo (37)	ENSG00000133048
PTN	AL565812	DOWN	Calvo (37)	ENSG00000105894
CALML3	M58026	DOWN	Calvo (37)	ENSG00000178363
IGFBP3	M31159	DOWN	Calvo (37)	ENSG00000146674
HPGD	J05594	DOWN	Calvo (37)	ENSG00000164120
PTN	BC005916	DOWN	Calvo (37)	ENSG00000105894
DCN	AF138303	DOWN	Calvo (37)	ENSG00000011465
DCN	AF138302	DOWN	Calvo (37)	ENSG00000011465
SNAI2	AI572079	DOWN	Calvo (37)	ENSG00000019549
PTPRD	N73931	DOWN	Calvo (37)	ENSG00000153707
NPTX2	U26662	DOWN	Calvo (37)	ENSG00000106236
KRT6B	AI831452	DOWN	Calvo (37)	ENSG00000185479
PTPRD	BF062299	DOWN	Calvo (37)	ENSG00000153707
DOK5	AL050069	DOWN	Calvo (37)	ENSG00000101134
GREM1	NM_013372	DOWN	Calvo (37)	ENSG00000166923
GREM1	NM_013372	DOWN	Calvo (37)	ENSG00000166923
EGFL6	NM_015507	DOWN	Calvo (37)	ENSG00000198759
PDPN	AU154455	DOWN	Calvo (37)	ENSG00000162493
EPHA3	AF213459	DOWN	Calvo (37)	ENSG00000044524
HTR2B	NM_000867	DOWN	Calvo (37)	ENSG00000135914
CD1A	M28825	DOWN	Calvo (37)	ENSG00000158477
DKK2	NM_014421	DOWN	Calvo (37)	ENSG00000155011
DDR1	U48705	DOWN	Calvo (37)	ENSG00000204580
CCL5	M21121	DOWN	Calvo (37)	ENSG00000271503
EPHB3	X75208	DOWN	Calvo (37)	ENSG00000182580
CYP2A6	M33318	DOWN	Calvo (37)	ENSG00000255974
MMP14	Z48481	DOWN	Calvo (37)	ENSG00000157227
PRPF8	NM_006445	DOWN	Calvo (37)	ENSG00000174231
EIF3S7	NM_003753	DOWN	Calvo (37)	ENSG00000100353
ATP6V0B	BC005876	DOWN	Calvo (37)	ENSG00000117410
MSN	NM_002444	DOWN	Calvo (37)	ENSG00000147065
DSP	NM_004415	DOWN	Calvo (37)	ENSG00000096696
PTPRF	AU145351	DOWN	Calvo (37)	ENSG00000142949
PTPRF	NM_002840	DOWN	Calvo (37)	ENSG00000142949
PTPRF	AI762627	DOWN	Calvo (37)	ENSG00000142949
S100A11	NM_005620	DOWN	Calvo (37)	ENSG00000163191
SPARC	NM_003118	DOWN	Calvo (37)	ENSG00000113140
XBP1	NM_005080	DOWN	Calvo (37)	ENSG00000100219
SPTBN1	N92501	DOWN	Calvo (37)	ENSG00000115306
SPTBN1	NM_003128	DOWN	Calvo (37)	ENSG00000115306
SFRS11	AU146237	DOWN	Calvo (37)	ENSG00000116754
KDELR2	AL542253	DOWN	Calvo (37)	ENSG00000136240
KDELR2	AL542253	DOWN	Calvo (37)	ENSG00000136240
SKP1A	BE964043	DOWN	Calvo (37)	ENSG00000113558
CALU	BF939365	DOWN	Calvo (37)	ENSG00000128595
CALU	U67280	DOWN	Calvo (37)	ENSG00000128595
CALU	NM_001219	DOWN	Calvo (37)	ENSG00000128595
ARL6IP5	N92494	DOWN	Calvo (37)	ENSG00000144746
CTNNA1	AI826881	DOWN	Calvo (37)	ENSG00000044115
CTNNA1	NM_001903	DOWN	Calvo (37)	ENSG00000044115
CTSD	NM_001909	DOWN	Calvo (37)	ENSG00000117984
ODC1	NM_002539	DOWN	Calvo (37)	ENSG00000115758
HSPA1A	NM_005345	DOWN	Calvo (37)	ENSG00000204389
PLOD1	NM_000302	DOWN	Calvo (37)	ENSG00000083444
SCD	AA678241	DOWN	Calvo (37)	ENSG00000099194

SCD	AB032261	DOWN	Calvo (37)	ENSG00000099194
DHCR24	NM_014762	DOWN	Calvo (37)	ENSG00000116133
CKB	NM_001823	DOWN	Calvo (37)	ENSG00000166165
PPM1J	NM_005167	DOWN	Calvo (37)	ENSG00000155367
FKBP4	NM_002014	DOWN	Calvo (37)	ENSG00000004478
FKBP4	NM_002014	DOWN	Calvo (37)	ENSG00000004478
PALLD	NM_016081	DOWN	Calvo (37)	ENSG00000129116
MGEA5	AK002091	DOWN	Calvo (37)	ENSG00000198408
HLA-E	X56841	DOWN	Calvo (37)	ENSG00000204592
HLA-E	NM_005516	DOWN	Calvo (37)	ENSG00000204592
PALLD	NM_016081	DOWN	Calvo (37)	ENSG00000129116
PALLD	AU157932	DOWN	Calvo (37)	ENSG00000129116
TACC1	NM_006283	DOWN	Calvo (37)	ENSG00000147526
BTG1	AL535380	DOWN	Calvo (37)	ENSG00000133639
BTG1	NM_001731	DOWN	Calvo (37)	ENSG00000133639
LGALS3BP	NM_005567	DOWN	Calvo (37)	ENSG00000108679
HSBP1	AK026575	DOWN	Calvo (37)	ENSG00000230989
HSBP1	NM_001537	DOWN	Calvo (37)	ENSG00000230989
CCND2	AI635187	DOWN	Calvo (37)	ENSG00000118971
CCND2	NM_001759	DOWN	Calvo (37)	ENSG00000118971
TBC1D8	AI348010	DOWN	Calvo (37)	ENSG00000204634
ABLIM1	NM_006720	DOWN	Calvo (37)	ENSG00000099204
ANXA6	NM_001155	DOWN	Calvo (37)	ENSG00000197043
CKAP4	AW029619	DOWN	Calvo (37)	ENSG00000136026
CKAP4	NM_006825	DOWN	Calvo (37)	ENSG00000136026
CD9	NM_001769	DOWN	Calvo (37)	ENSG00000010278
TXNIP	NM_006472	DOWN	Calvo (37)	ENSG00000265972
TXNIP	NM_006472	DOWN	Calvo (37)	ENSG00000265972
TXNIP	NM_006472	DOWN	Calvo (37)	ENSG00000265972
JUP	NM_021991	DOWN	Calvo (37)	ENSG00000173801
DSTN	BF697964	DOWN	Calvo (37)	ENSG00000125868
DSTN	BF697964	DOWN	Calvo (37)	ENSG00000125868
LDHB	NM_002300	UP	Calvo (37)	ENSG00000111716
ADD3	BE545756	UP	Calvo (37)	ENSG00000148700
CTTN	NM_005231	UP	Calvo (37)	ENSG00000085733
LGALS1	NM_002305	UP	Calvo (37)	ENSG00000100097
THBS1	AI812030	UP	Calvo (37)	ENSG00000137801
THBS1	NM_003246	UP	Calvo (37)	ENSG00000137801
THBS1	AV726673	UP	Calvo (37)	ENSG00000137801
THBS1	NM_003246	UP	Calvo (37)	ENSG00000137801
ITGB5	AL048423	UP	Calvo (37)	ENSG00000082781
ITGB5	NM_002213	UP	Calvo (37)	ENSG00000082781
CDH1	NM_004360	UP	Calvo (37)	ENSG00000039068
CDH1	NM_004360	UP	Calvo (37)	ENSG00000039068
HLA-DPB1	NM_002121	UP	Calvo (37)	ENSG00000223865
GPNMB	NM_002510	UP	Calvo (37)	ENSG00000136235
TIMP3	NM_000362	UP	Calvo (37)	ENSG00000100234
MBNL1	BF512200	UP	Calvo (37)	ENSG00000152601
MBNL1	N31913	UP	Calvo (37)	ENSG00000152601
MBNL1	N31913	UP	Calvo (37)	ENSG00000152601
HTRA1	NM_002775	UP	Calvo (37)	ENSG00000166033
DDX3X	NM_001356	UP	Calvo (37)	ENSG00000215301
PLS3	NM_005032	UP	Calvo (37)	ENSG00000102024
CTBP2	N23018	UP	Calvo (37)	ENSG00000175029
ZRANB1	AW269836	UP	Calvo (37)	ENSG00000019995
CTBP2	NM_001329	UP	Calvo (37)	ENSG00000175029
ATP1B1	BC000006	UP	Calvo (37)	ENSG00000143153
ATP1B1	NM_001677	UP	Calvo (37)	ENSG00000143153
SYPL1	AI768845	UP	Calvo (37)	ENSG00000008282
BGN	BC002416	UP	Calvo (37)	ENSG00000182492
BGN	BC002416	UP	Calvo (37)	ENSG00000182492
SDC1	NM_002997	UP	Calvo (37)	ENSG00000115884
ARHGDIB	NM_001175	UP	Calvo (37)	ENSG00000111348
CYR61	NM_001554	UP	Calvo (37)	ENSG00000142871
MOBK1B	NM_018221	UP	Calvo (37)	ENSG00000114978
ANP32B	NM_006401	UP	Calvo (37)	ENSG00000136938
C5orf13	NM_004772	UP	Calvo (37)	ENSG00000134986
C5orf13	NM_004772	UP	Calvo (37)	ENSG00000134986
EMP1	NM_001423	UP	Calvo (37)	ENSG00000134531
EMP1	NM_001423	UP	Calvo (37)	ENSG00000134531
ETS2	AL575509	UP	Calvo (37)	ENSG00000157557
ETS2	NM_005239	UP	Calvo (37)	ENSG00000157557
ENC1	AF010314	UP	Calvo (37)	ENSG00000171617
ENC1	NM_003633	UP	Calvo (37)	ENSG00000171617
SLC9A3R1	NM_004252	UP	Calvo (37)	ENSG00000109062
ZFP36L2	AI356398	UP	Calvo (37)	ENSG00000152518
ZFP36L2	AI356398	UP	Calvo (37)	ENSG00000152518
ZFP36L2	NM_006887	UP	Calvo (37)	ENSG00000152518
UCHL1	NM_004181	UP	Calvo (37)	ENSG00000154277
ADRBK1	M80776	UP	Calvo (37)	ENSG00000173020
MGST3	NM_004528	UP	Calvo (37)	ENSG00000143198
SOX4	NM_003107	UP	Calvo (37)	ENSG00000124766
SOX4	AL136179	UP	Calvo (37)	ENSG00000124766
SOX4	NM_003107	UP	Calvo (37)	ENSG00000124766
CLDN4	NM_001305	UP	Calvo (37)	ENSG00000189143
DPYSL3	NM_001387	UP	Calvo (37)	ENSG00000113657

COL6A3	NM_004369	UP	Calvo (37)	ENSG00000163359
NQO1	AI039874	UP	Calvo (37)	ENSG00000181019
NQO1	NM_000903	UP	Calvo (37)	ENSG00000181019
CTSC	NM_001814	UP	Calvo (37)	ENSG00000109861
PRCP	NM_005040	UP	Calvo (37)	ENSG00000137509
LAMB1	NM_002291	UP	Calvo (37)	ENSG00000091136
IGFBP4	NM_001552	UP	Calvo (37)	ENSG00000141753
ELF3	AF017307	UP	Calvo (37)	ENSG00000163435
RPA1	NM_002945	UP	Calvo (37)	ENSG00000132383
RPA1	NM_002945	UP	Calvo (37)	ENSG00000132383
FHL1	U29538	UP	Calvo (37)	ENSG00000022267
FHL1	NM_001449	UP	Calvo (37)	ENSG00000022267
SAR1A	NM_020150	UP	Calvo (37)	ENSG00000079332
JARID1B	NM_006618	UP	Calvo (37)	ENSG00000117139
SORD	L29008	UP	Calvo (37)	ENSG00000140263
SORD	L29008	UP	Calvo (37)	ENSG00000140263
FSCN1	NM_003088	UP	Calvo (37)	ENSG00000075618
NME1	NM_000269	UP	Calvo (37)	ENSG00000239672
FAT	NM_005245	UP	Calvo (37)	ENSG00000083857
ANXA2	NM_004039	UP	Calvo (37)	ENSG00000182718
KRT18	NM_000224	UP	Calvo (37)	ENSG00000111057
CNN2	NM_004368	UP	Calvo (37)	ENSG00000064666
NBL1	NM_005380	UP	Calvo (37)	ENSG00000158747
IER3	NM_003897	UP	Calvo (37)	ENSG00000137331
RAVER2	AL039831	UP	Calvo (37)	ENSG00000162437
KRT19	NM_002276	UP	Calvo (37)	ENSG00000171345
ACSL3	AL525798	UP	Calvo (37)	ENSG00000123983
ACSL3	D89053	UP	Calvo (37)	ENSG00000123983
ACSL3	D89053	UP	Calvo (37)	ENSG00000123983
TIMP1	NM_003254	UP	Calvo (37)	ENSG00000102265
GJA1	NM_000165	UP	Calvo (37)	ENSG00000152661
DLG5	AB011155	UP	Calvo (37)	ENSG00000151208
TPD52	NM_005079	UP	Calvo (37)	ENSG00000076554
TPD52	AA524023	UP	Calvo (37)	ENSG00000076554
TPD52	NM_005079	UP	Calvo (37)	ENSG00000076554
CCND3	NM_001760	UP	Calvo (37)	ENSG00000112576
ENTPD6	NM_001247	UP	Calvo (37)	ENSG00000197586
TUBG1	NM_001070	UP	Calvo (37)	ENSG00000131462
EPB41L2	BF511685	UP	Calvo (37)	ENSG00000079819
EPB41L2	NM_001431	UP	Calvo (37)	ENSG00000079819
LAPTM5	AI589086	UP	Calvo (37)	ENSG00000162511
LAPTM5	NM_006762	UP	Calvo (37)	ENSG00000162511
LUM	NM_002345	UP	Calvo (37)	ENSG00000139329
PTK9	NM_002822	UP	Calvo (37)	ENSG00000151239
ADD3	AI763123	UP	Calvo (37)	ENSG00000148700
ADD3	NM_019903	UP	Calvo (37)	ENSG00000148700
COX6C	NM_004374	UP	Calvo (37)	ENSG00000164919
MCM5	NM_006739	UP	Calvo (37)	ENSG00000100297
TMEM106C	NM_024056	UP	Calvo (37)	ENSG00000134291
AIP	AL558532	UP	Calvo (37)	ENSG00000110711
AIP	NM_003977	UP	Calvo (37)	ENSG00000110711
FBLN1	NM_001996	UP	Calvo (37)	ENSG00000077942
DHCR7	AW150953	UP	Calvo (37)	ENSG00000172893
DHCR7	NM_001360	UP	Calvo (37)	ENSG00000172893
AEBP1	NM_001129	UP	Calvo (37)	ENSG00000106624
LBR	NM_002296	UP	Calvo (37)	ENSG00000143815
FER1L3	NM_013451	UP	Calvo (37)	ENSG00000138119
SH3BP5	NM_004844	UP	Calvo (37)	ENSG00000131370
TOMM7	NM_019059	UP	Calvo (37)	ENSG00000196683
TIMM17A	NM_006335	UP	Calvo (37)	ENSG00000134375
RNF14	AB022663	UP	Calvo (37)	ENSG00000013561
SCCPDH	NM_016002	UP	Calvo (37)	ENSG00000143653
TACSTD1	NM_002354	UP	Calvo (37)	ENSG00000119888
HSPB1	NM_001540	UP	Calvo (37)	ENSG00000106211
EFEMP1	NM_004105	UP	Calvo (37)	ENSG00000115380
BNIP3	U15174	UP	Calvo (37)	ENSG00000176171
BNIP3	NM_004052	UP	Calvo (37)	ENSG00000176171
SFRP2	AF311912	DOWN	Feng (38)	ENSG00000145423
COL11A1	NM_001854	DOWN	Feng (38)	ENSG00000060718
PDGFRL	NM_006207	DOWN	Feng (38)	ENSG00000104213
MMP2	NM_004530	DOWN	Feng (38)	ENSG00000087245
CTSK	NM_000396	DOWN	Feng (38)	ENSG00000143387
MMP7	NM_002423	DOWN	Feng (38)	ENSG00000137673
ASPN	NM_017680	DOWN	Feng (38)	ENSG00000106819
ITGBL1	NM_004791	DOWN	Feng (38)	ENSG00000198542
OSF-2	NM_006475	DOWN	Feng (38)	ENSG00000133110
COL6A1	NM_001848	DOWN	Feng (38)	ENSG00000142156
FMO1	NM_002021	DOWN	Feng (38)	ENSG00000010932
COL5A2	NM_000393	DOWN	Feng (38)	ENSG00000204262
HTRA3	AY040094	DOWN	Feng (38)	ENSG00000170801
SPOCK	NM_004598	DOWN	Feng (38)	ENSG00000152377
LUM	NM_002345	DOWN	Feng (38)	ENSG00000139329
SPON1	AB018305	DOWN	Feng (38)	ENSG00000262655
CILP	NM_003613	DOWN	Feng (38)	ENSG00000138615
PTPRC	NM_002838	DOWN	Feng (38)	ENSG00000081237
LGALS1	NM_002305	DOWN	Feng (38)	ENSG00000100097

PRSS11	NM_002775	DOWN	Feng (38)	ENSG00000166033
OGN	NM_033014	DOWN	Feng (38)	ENSG00000106809
ECM1	NM_004425	DOWN	Feng (38)	ENSG00000143369
STEAP	NM_012449	DOWN	Feng (38)	ENSG00000164647
SNAI2	BC014890	DOWN	Feng (38)	ENSG00000019549
FN1	NM_002026	DOWN	Feng (38)	ENSG00000115414
TNFAIP6	NM_007115	DOWN	Feng (38)	ENSG00000123610
COL1A2	NM_000089	DOWN	Feng (38)	ENSG00000164692
COL3A1	NM_000090	DOWN	Feng (38)	ENSG00000168542
FBN1	NM_000138	DOWN	Feng (38)	ENSG00000166147
CCL8	Y16645	DOWN	Feng (38)	ENSG00000108700
CALU	NM_001219	DOWN	Feng (38)	ENSG00000128595
CSPG2	U16306	DOWN	Feng (38)	ENSG00000038427
GPNMB	NM_002510	DOWN	Feng (38)	ENSG00000136235
RUNX2	AW469546	DOWN	Feng (38)	ENSG00000124813
GRP	NM_002091	DOWN	Feng (38)	ENSG00000134443
PCOLCE	NM_002593	DOWN	Feng (38)	ENSG00000106333
TGFB3	NM_003239	DOWN	Feng (38)	ENSG00000119699
COL5A1	NM_000093	DOWN	Feng (38)	ENSG00000130635
CDC45L	NM_003504	DOWN	Feng (38)	ENSG00000093009
KRT14	NM_000526	DOWN	Feng (38)	ENSG00000186847
CTHRC1	BC014245	DOWN	Feng (38)	ENSG00000164932
RAFTLIN	D42043	DOWN	Feng (38)	ENSG00000131378
MRC2	NM_006039	DOWN	Feng (38)	ENSG00000011028
RIS1	AF438313	DOWN	Feng (38)	ENSG00000249992
CORO1A	NM_007074	DOWN	Feng (38)	ENSG00000102879
FCGR3A	J04162	DOWN	Feng (38)	ENSG00000203747
FOXO1A	NM_002015	DOWN	Feng (38)	ENSG00000150907
CR2	NM_001877	DOWN	Feng (38)	ENSG00000117322
TXNIP	NM_006472	DOWN	Feng (38)	ENSG00000265972
CD48	NM_001778	DOWN	Feng (38)	ENSG00000117091
PDK4	AF334710	DOWN	Feng (38)	ENSG00000004799
ABCC1	NM_004996	DOWN	Feng (38)	ENSG00000103222
PRG1	NM_002727	DOWN	Feng (38)	ENSG00000122862
CKTSF1B1	NM_013372	DOWN	Feng (38)	ENSG00000276886
PDE6H	NM_006205 22	UP	Feng (38)	ENSG00000139053
MS4A1	NM_021950 21	UP	Feng (38)	ENSG00000156738
C7	NM_000587 21	UP	Feng (38)	ENSG00000112936
LTB	NM_002341 18	UP	Feng (38)	ENSG00000227507
SELL	NM_000655 18	UP	Feng (38)	ENSG00000188404
RGS1	NM_002922 18	UP	Feng (38)	ENSG00000090104
FCRLA	NM_032738 17	UP	Feng (38)	ENSG00000132185
BIRC3	AF070674	UP	Feng (38)	ENSG00000023445
TNFAIP3	NM_006290 17	UP	Feng (38)	ENSG00000118503
DUSP2	NM_004418 16	UP	Feng (38)	ENSG00000158050
ZFP36L2	U07802	UP	Feng (38)	ENSG00000152518
HBB	NM_000518 15	UP	Feng (38)	ENSG00000244734
TCL1A	NM_021966 15	UP	Feng (38)	ENSG00000100721
KLF2	NM_016270 15	UP	Feng (38)	ENSG00000127528
GPR183	NM_004951 15	UP	Feng (38)	ENSG00000169508
KRT15	NM_002275 15	UP	Feng (38)	ENSG00000171346
CCL19	NM_006274 15	UP	Feng (38)	ENSG00000172724
NEFL	NM_006158 14	UP	Feng (38)	ENSG00000277586
PIGR	AK026320	UP	Feng (38)	ENSG00000162896
	NM_003726	UP	Hao (39)	ENSG00000141293
	NM_002382	UP	Hao (39)	ENSG00000125952
	NM_005000	UP	Hao (39)	ENSG00000128609
	NM_005050	UP	Hao (39)	ENSG00000119688
	NM_004431	UP	Hao (39)	ENSG00000142627
	NM_003355	UP	Hao (39)	ENSG00000175567
	NM_004417	UP	Hao (39)	ENSG00000120129
	NM_001406	UP	Hao (39)	ENSG00000108947
	NM_003118	DOWN	Hao (39)	ENSG00000113140
	NM_000422	DOWN	Hao (39)	ENSG00000128422
	NM_006475	DOWN	Hao (39)	ENSG00000133110
	NM_006435	DOWN	Hao (39)	ENSG00000185201
	NM_021034	DOWN	Hao (39)	ENSG00000142089
	NM_003507	DOWN	Hao (39)	ENSG00000155760
	NM_003255	DOWN	Hao (39)	ENSG00000035862
	NM_005507	DOWN	Hao (39)	ENSG00000172757
	NM_006745	DOWN	Hao (39)	ENSG00000052802
	NM_002731	DOWN	Hao (39)	ENSG00000142875
	NM_002211	DOWN	Hao (39)	ENSG00000150093
	NM_000362	DOWN	Hao (39)	ENSG00000100234
	NM_003000	DOWN	Hao (39)	ENSG00000117118
	NM_001854.1	DOWN	Suzuki (44)	ENSG00000060718
	NM_017680.1	DOWN	Suzuki (44)	ENSG00000106819
	NM_002427.2	DOWN	Suzuki (44)	ENSG00000137745
	NM_002421.2	DOWN	Suzuki (44)	ENSG00000196611
	NM_002048.1	DOWN	Suzuki (44)	ENSG00000180447
	NM_002423.2	DOWN	Suzuki (44)	ENSG00000137673
	NM_006207.1	DOWN	Suzuki (44)	ENSG00000104213
	NM_013989.1	DOWN	Suzuki (44)	ENSG00000211448
	NM_004791.1	DOWN	Suzuki (44)	ENSG00000198542
	NM_001609.1	DOWN	Suzuki (44)	ENSG00000196177
	NM_002422.2	DOWN	Suzuki (44)	ENSG00000149968

	NM_001548.1	DOWN	Suzuki (44)	ENSG00000185745
	NM_006486.1	DOWN	Suzuki (44)	ENSG00000077942
	NM_000396.1	DOWN	Suzuki (44)	ENSG00000143387
	NM_000129.2	DOWN	Suzuki (44)	ENSG00000124491
	NM_014333.1	DOWN	Suzuki (44)	ENSG00000182985
	NM_006307.1	DOWN	Suzuki (44)	ENSG00000101955
	NM_004530.1	DOWN	Suzuki (44)	ENSG00000087245
	NM_004684.1	DOWN	Suzuki (44)	ENSG00000152583
	NM_002345.1	DOWN	Suzuki (44)	ENSG00000139329
	NM_000138.1	DOWN	Suzuki (44)	ENSG00000166147
	NM_004207.1	DOWN	Suzuki (44)	ENSG00000141526
	NM_001038.1	DOWN	Suzuki (44)	ENSG00000111319
	NM_006868.1	DOWN	Suzuki (44)	ENSG00000168461
	NM_000089.1	DOWN	Suzuki (44)	ENSG00000164692
	NM_014840.1	DOWN	Suzuki (44)	ENSG00000074590
	NM_000393.1	DOWN	Suzuki (44)	ENSG00000204262
	NM_012449.1	DOWN	Suzuki (44)	ENSG00000164647
	NM_004566.1	DOWN	Suzuki (44)	ENSG00000170525
	NM_019058.1	DOWN	Suzuki (44)	ENSG00000168209
	NM_016938.1	DOWN	Suzuki (44)	ENSG00000172638
	NM_015675.1	DOWN	Suzuki (44)	ENSG00000099860
	NM_018845.1	DOWN	Suzuki (44)	ENSG00000169241
	NM_004508.1	DOWN	Suzuki (44)	ENSG00000067064
	NM_012072.2	DOWN	Suzuki (44)	ENSG00000125810
	NM_000611.1	DOWN	Suzuki (44)	ENSG00000085063
	NM_013943.1	DOWN	Suzuki (44)	ENSG00000169504
	NM_018097.1	DOWN	Suzuki (44)	ENSG00000137814
	NM_004768.1	DOWN	Suzuki (44)	ENSG00000116754
	NM_004199.1	DOWN	Suzuki (44)	ENSG00000072682
	NM_015626.1	DOWN	Suzuki (44)	ENSG00000109046
	NM_000521.2	DOWN	Suzuki (44)	ENSG00000049860
	NM_016246.1	DOWN	Suzuki (44)	ENSG00000087076
	NM_006419.1	UP	Suzuki (44)	ENSG00000156234
	NM_000587.1	UP	Suzuki (44)	ENSG00000112936
	NM_002341.1	UP	Suzuki (44)	ENSG00000227507
	NM_016459.1	UP	Suzuki (44)	ENSG00000170476
	NM_003385.1	UP	Suzuki (44)	ENSG00000163032
	NM_000954.1	UP	Suzuki (44)	ENSG00000107317
	NM_003933.2	UP	Suzuki (44)	ENSG00000007516
	NM_007286.1	UP	Suzuki (44)	ENSG00000171992
	NM_003057.1	UP	Suzuki (44)	ENSG00000175003
	NM_001803.1	UP	Suzuki (44)	ENSG00000169442
	NM_016418.1	UP	Suzuki (44)	ENSG00000186575
	NM_001536.1	UP	Suzuki (44)	ENSG00000126457
	NM_003220.1	UP	Suzuki (44)	ENSG00000137203
	NM_007153.1	UP	Suzuki (44)	ENSG00000160321
	NM_000206.1	UP	Suzuki (44)	ENSG00000147168
	NM_003604.1	UP	Suzuki (44)	ENSG00000133124
	NM_003254.1	UP	Suzuki (44)	ENSG00000102265
ASPN	NM_017680	DOWN	Ellsworth (42)	ENSG00000106819
COL11A1	NM_001854	DOWN	Ellsworth (42)	ENSG00000060718
CNIH3	NM_152495	DOWN	Ellsworth (42)	ENSG00000143786
FST	NM_013409	DOWN	Ellsworth (42)	ENSG00000134363
GRP	NM_002091	DOWN	Ellsworth (42)	ENSG00000134443
KRT14	NM_000526	DOWN	Ellsworth (42)	ENSG00000186847
KERA	NM_007035	DOWN	Ellsworth (42)	ENSG00000139330
LRRC15	NM_130830	DOWN	Ellsworth (42)	ENSG00000172061
MRC2	NM_006039	DOWN	Ellsworth (42)	ENSG00000011028
MMP10	NM_002425	DOWN	Ellsworth (42)	ENSG00000166670
MMP13	NM_002427	DOWN	Ellsworth (42)	ENSG00000137745
MMP16	NM_005941	DOWN	Ellsworth (42)	ENSG00000156103
MXRA5	NM_015419	DOWN	Ellsworth (42)	ENSG00000101825
MME	NM_000902	DOWN	Ellsworth (42)	ENSG00000196549
MFAP5	NM_003480	DOWN	Ellsworth (42)	ENSG00000197614
NRG1	NM_013957	DOWN	Ellsworth (42)	ENSG00000157168
OGN	NM_014057	DOWN	Ellsworth (42)	ENSG00000106809
POSTN	NM_007257	DOWN	Ellsworth (42)	ENSG00000133110
RBP4	NM_006744	DOWN	Ellsworth (42)	ENSG00000138207
SCG5	NM_003020	DOWN	Ellsworth (42)	ENSG00000166922
SPON1	NM_006108	DOWN	Ellsworth (42)	ENSG00000262655
SPON2	NM_012445	DOWN	Ellsworth (42)	ENSG00000159674
TAC1	NM_003182	DOWN	Ellsworth (42)	ENSG00000006128
TNN	NM_022093	DOWN	Ellsworth (42)	ENSG00000120332
TNFAIP6	NM_007115	DOWN	Ellsworth (42)	ENSG00000123610
WT1	NM_024426	DOWN	Ellsworth (42)	ENSG00000184937
WNT2	NM_003391	DOWN	Ellsworth (42)	ENSG00000105989
ART4	NM_021071	UP	Ellsworth (42)	ENSG00000111339
C7	NM_000587	UP	Ellsworth (42)	ENSG00000112936
CCDC102B	NM_024781	UP	Ellsworth (42)	ENSG00000150636
CCL21	NM_002989	UP	Ellsworth (42)	ENSG00000137077
CD79B	NM_000626	UP	Ellsworth (42)	ENSG00000007312
COL4A4	NM_000092	UP	Ellsworth (42)	ENSG00000081052
DNASE1L3	NM_004944	UP	Ellsworth (42)	ENSG00000163687
EPHA3	NM_005233	UP	Ellsworth (42)	ENSG00000044524
FOXF1	NM_001451	UP	Ellsworth (42)	ENSG00000103241
FOXG1	NM_005249	UP	Ellsworth (42)	ENSG00000176165

CR2	NM_001877	UP	Vecchi (43)	ENSG00000117322
P2RX5	U49396	UP	Vecchi (43)	ENSG00000083454
EPHA3	AF213459	UP	Vecchi (43)	ENSG00000044524
MS4A1	AW474852	UP	Vecchi (43)	ENSG00000156738
BANK1	NM_017935	UP	Vecchi (43)	ENSG00000153064
SLC26A7	AI758950	UP	Vecchi (43)	ENSG00000147606
PAX5	BF510692	UP	Vecchi (43)	ENSG00000196092
C4orf7	AI718421	UP	Vecchi (43)	ENSG00000181617
MS4A1	AI808597	UP	Vecchi (43)	ENSG00000156738
FCRH1	AI572979	UP	Vecchi (43)	ENSG00000163534
T3JAM	NM_025228	UP	Vecchi (43)	ENSG00000009790
POU2AF1	NM_006235	UP	Vecchi (43)	ENSG00000110777
BCL11A	NM_022893	UP	Vecchi (43)	ENSG00000119866
MS4A1	X12530	UP	Vecchi (43)	ENSG00000156738
MS4A1	BC002807	UP	Vecchi (43)	ENSG00000156738
GPR18	AF261135	UP	Vecchi (43)	ENSG00000125245
C7	NM_000587	UP	Vecchi (43)	ENSG00000112936
	T57776	UP	Vecchi (43)	ENSG00000111339
BRDG1	NM_012108	UP	Vecchi (43)	ENSG00000035720
CCL19	U88321	UP	Vecchi (43)	ENSG00000172724
BCL11A	AI912275	UP	Vecchi (43)	ENSG00000119866
TRIM	AJ240085	UP	Vecchi (43)	ENSG00000163519
MS4A1	AI862674	UP	Vecchi (43)	ENSG00000156738
SELL	NM_000655	UP	Vecchi (43)	ENSG00000188404
GPR64	NM_005756	UP	Vecchi (43)	ENSG00000173698
LRMP	U10485	UP	Vecchi (43)	ENSG00000118308
	AW292872	UP	Vecchi (43)	ENSG00000271856
COL4A3	AI694562	UP	Vecchi (43)	ENSG00000169031
BCL11B	AA918317	UP	Vecchi (43)	ENSG00000127152
IGHM	X17115	UP	Vecchi (43)	ENSG00000211899
CDW52	N90866	UP	Vecchi (43)	ENSG00000169442
TOSO	AI084226	UP	Vecchi (43)	ENSG00000162894
H963	NM_013308	UP	Vecchi (43)	ENSG00000174946
HLA-DOB	NM_002120	UP	Vecchi (43)	ENSG00000241106
ZNFN1A1	AI741188	UP	Vecchi (43)	ENSG00000185811
BCL11A	NM_018014	UP	Vecchi (43)	ENSG00000119866
KIAA0746	AB018289	UP	Vecchi (43)	ENSG00000091490
IL7R	NM_002185	UP	Vecchi (43)	ENSG00000168685
IL7R	BE217880	UP	Vecchi (43)	ENSG00000168685
PSCDBP	L06633	UP	Vecchi (43)	ENSG00000115165
MEOX2	NM_005924	UP	Vecchi (43)	ENSG00000106511
TAGAP	AW576600	UP	Vecchi (43)	ENSG00000164691
T3JAM	AL022398	UP	Vecchi (43)	ENSG00000009790
PTPRC	AI809341	UP	Vecchi (43)	ENSG00000081237
PTPRC	Y00062	UP	Vecchi (43)	ENSG00000081237
PLAC8	NM_016619	UP	Vecchi (43)	ENSG00000145287
FCRH3	BF514552	UP	Vecchi (43)	ENSG00000160856
ATP8A1	AI769688	UP	Vecchi (43)	ENSG00000124406
LAMP3	NM_014398	UP	Vecchi (43)	ENSG00000078081
CXCR4	L01639	UP	Vecchi (43)	ENSG00000121966
SERPINB9	BC002538	UP	Vecchi (43)	ENSG00000170542
LCK	NM_005356	UP	Vecchi (43)	ENSG00000182866
HA-1	BE349017	UP	Vecchi (43)	ENSG00000180448
BIRC3	U37546	UP	Vecchi (43)	ENSG00000023445
CCR7	NM_001838	UP	Vecchi (43)	ENSG00000126353
MMP9	NM_004994	UP	Vecchi (43)	ENSG00000100985
CSF2RB	AV756141	UP	Vecchi (43)	ENSG00000100368
CYFIP2	AL161999	UP	Vecchi (43)	ENSG00000055163
LAT	AF036906	UP	Vecchi (43)	ENSG00000213658
MST4	NM_016542	UP	Vecchi (43)	ENSG00000134602
VCAM1	NM_001078	UP	Vecchi (43)	ENSG00000162692
TNFAIP8	BC005352	UP	Vecchi (43)	ENSG00000145779
RAC2	BE138888	UP	Vecchi (43)	ENSG00000128340
RASGRP1	NM_005739	UP	Vecchi (43)	ENSG00000172575
PRKCB1	M13975	UP	Vecchi (43)	ENSG00000166501
CD3D	NM_000732	UP	Vecchi (43)	ENSG00000167286
LY75	NM_002349	UP	Vecchi (43)	ENSG00000054219
MAL	NM_002371	UP	Vecchi (43)	ENSG00000172005
STK17B	NM_004226	UP	Vecchi (43)	ENSG00000081320
EVI2B	BC005926	UP	Vecchi (43)	ENSG00000185862
SLC39A10	AI700476	UP	Vecchi (43)	ENSG00000196950
TNFAIP8	NM_014350	UP	Vecchi (43)	ENSG00000145779
PDE3B	NM_000753	UP	Vecchi (43)	ENSG00000152270
RAPGEF6	NM_016340	UP	Vecchi (43)	ENSG00000158987
TAGAP	BF591040	UP	Vecchi (43)	ENSG00000164691
CXCR4	AJ224869	UP	Vecchi (43)	ENSG00000121966
PLCG2	NM_002661	UP	Vecchi (43)	ENSG00000197943
FNBP1	AB011126	UP	Vecchi (43)	ENSG00000187239
TAP2	AA573502	UP	Vecchi (43)	ENSG00000204267
SIAT1	AI743792	UP	Vecchi (43)	ENSG00000073849
RAPGEF6	AI640834	UP	Vecchi (43)	ENSG00000158987
ICSBP1	AI073984	UP	Vecchi (43)	ENSG00000140968
ETS1	BE218980	UP	Vecchi (43)	ENSG00000134954
CENTD1	AB011152	UP	Vecchi (43)	ENSG00000047365
PLEKHA2	BF968578	UP	Vecchi (43)	ENSG00000169499
ITGA8	AI193623	UP	Vecchi (43)	ENSG00000077943

ADRBK2	AI651212	UP	Vecchi (43)	ENSG00000100077
SATB1	NM_002971	UP	Vecchi (43)	ENSG00000182568
GLS	NM_014905	UP	Vecchi (43)	ENSG00000115419
CD53	NM_000560	UP	Vecchi (43)	ENSG00000143119
BLNK	NM_013314	UP	Vecchi (43)	ENSG00000095585
TCF3	M31523	UP	Vecchi (43)	ENSG00000071564
AP1GBP1	AI472320	UP	Vecchi (43)	ENSG00000275066
TNFAIP3	NM_006290	UP	Vecchi (43)	ENSG00000118503
FLJ10652	NM_018169	UP	Vecchi (43)	ENSG00000174718
CD83	NM_004233	UP	Vecchi (43)	ENSG00000112149
LOC64744	AL137764	UP	Vecchi (43)	ENSG00000084070
SNARK	NM_030952	UP	Vecchi (43)	ENSG00000163545
FCHSD2	NM_014824	UP	Vecchi (43)	ENSG00000137478
CD48	NM_001778	UP	Vecchi (43)	ENSG00000117091
ARHGEF18	AB011093	UP	Vecchi (43)	ENSG00000104880
FLJ22570	BC004564	UP	Vecchi (43)	ENSG00000146094
ZBTB24	NM_014797	UP	Vecchi (43)	ENSG00000112365
CSK	NM_004383	UP	Vecchi (43)	ENSG00000103653
LPXN	X77598	UP	Vecchi (43)	ENSG00000110031
MBNL1	NM_021038	UP	Vecchi (43)	ENSG00000152601
AKAP2	NM_007203	UP	Vecchi (43)	ENSG00000241978
MBNL1	N31913	UP	Vecchi (43)	ENSG00000152601
TP73L	AF091627	DOWN	Vecchi (43)	ENSG00000073282
MRC2	AB014609	DOWN	Vecchi (43)	ENSG00000011028
ANXA1	NM_000700	DOWN	Vecchi (43)	ENSG00000135046
COL3A1	AU144167	DOWN	Vecchi (43)	ENSG00000168542
FOXF2	NM_001452	DOWN	Vecchi (43)	ENSG00000137273
CDH11	D21254	DOWN	Vecchi (43)	ENSG00000140937
SPON2	NM_012445	DOWN	Vecchi (43)	ENSG00000159674
LGALS3	BC001120	DOWN	Vecchi (43)	ENSG00000131981
DKFZP434B044	AL136861	DOWN	Vecchi (43)	ENSG00000103196
COL5A1	AI130969	DOWN	Vecchi (43)	ENSG00000130635
IGFBP3	M31159	DOWN	Vecchi (43)	ENSG00000146674
FBXO32	N21643	DOWN	Vecchi (43)	ENSG00000156804
SPARC	NM_003118	DOWN	Vecchi (43)	ENSG00000113140
TAGLN	NM_003186	DOWN	Vecchi (43)	ENSG00000149591
FOXK2	AV763408	DOWN	Vecchi (43)	ENSG00000141568
PLXDC2	AI420817	DOWN	Vecchi (43)	ENSG00000120594
PLS3	NM_005032	DOWN	Vecchi (43)	ENSG00000102024
ACTA2	NM_001613	DOWN	Vecchi (43)	ENSG00000107796
SPARCL1	NM_004684	DOWN	Vecchi (43)	ENSG00000152583
TAZ	BF674349	DOWN	Vecchi (43)	ENSG00000102125
COL1A2	AA788711	DOWN	Vecchi (43)	ENSG00000164692
GEM	NM_005261	DOWN	Vecchi (43)	ENSG00000164949
NOX4	NM_016931	DOWN	Vecchi (43)	ENSG00000086991
GPNMB	NM_002510	DOWN	Vecchi (43)	ENSG00000136235
NNMT	NM_006169	DOWN	Vecchi (43)	ENSG00000166741
OLFML3	NM_020190	DOWN	Vecchi (43)	ENSG00000116774
SDC1	Z48199	DOWN	Vecchi (43)	ENSG00000115884
GULP1	AF200715	DOWN	Vecchi (43)	ENSG00000144366
PTN	M57399	DOWN	Vecchi (43)	ENSG00000105894
SPON1	AB018305	DOWN	Vecchi (43)	ENSG00000262655
GAS1	NM_002048	DOWN	Vecchi (43)	ENSG00000180447
GREM1	NM_013372	DOWN	Vecchi (43)	ENSG00000166923
C4A	K02403	DOWN	Vecchi (43)	ENSG00000244731
GPM6B	AF016004	DOWN	Vecchi (43)	ENSG00000046653
COL6A1	AA292373	DOWN	Vecchi (43)	ENSG00000142156
COL1A2	NM_000089	DOWN	Vecchi (43)	ENSG00000164692
SNAI2	AI572079	DOWN	Vecchi (43)	ENSG00000019549
COL3A1	AI813758	DOWN	Vecchi (43)	ENSG00000168542
TWIST1	X99268	DOWN	Vecchi (43)	ENSG00000122691
PCDH18	AW189885	DOWN	Vecchi (43)	ENSG00000189184
LRRC15	AU147799	DOWN	Vecchi (43)	ENSG00000172061
LUM	NM_002345	DOWN	Vecchi (43)	ENSG00000139329
CLDN11	AW264204	DOWN	Vecchi (43)	ENSG00000013297
ADAM12	NM_003474	DOWN	Vecchi (43)	ENSG00000148848
SPP1	M83248	DOWN	Vecchi (43)	ENSG00000118785
F13A1	NM_000129	DOWN	Vecchi (43)	ENSG00000124491
COL5A2	AL575735	DOWN	Vecchi (43)	ENSG00000204262
LOC253827	AW027333	DOWN	Vecchi (43)	ENSG00000174099
DCN	AI281593	DOWN	Vecchi (43)	ENSG00000011465
DKFZp564I1922	AF245505	DOWN	Vecchi (43)	ENSG00000101825
COL1A1	K01228	DOWN	Vecchi (43)	ENSG00000108821
CSPG2	NM_004385	DOWN	Vecchi (43)	ENSG00000038427
THBS2	NM_003247	DOWN	Vecchi (43)	ENSG00000186340
KIT	NM_000222	DOWN	Vecchi (43)	ENSG00000157404
PGDS	NM_014485	DOWN	Vecchi (43)	ENSG00000163106
COL5A2	NM_000393	DOWN	Vecchi (43)	ENSG00000204262
OMD	AI765819	DOWN	Vecchi (43)	ENSG00000127083
URB	AW303375	DOWN	Vecchi (43)	ENSG0000009198
FBLN1	NM_006486	DOWN	Vecchi (43)	ENSG00000077942
FN1	BC005858	DOWN	Vecchi (43)	ENSG00000115414
CTGF	M92934	DOWN	Vecchi (43)	ENSG00000118523
DCN	AF138300	DOWN	Vecchi (43)	ENSG00000011465
AKR1C3	AB018580	DOWN	Vecchi (43)	ENSG00000196139
LUM	AI141861	DOWN	Vecchi (43)	ENSG00000139329

FN1	X02761	DOWN	Vecchi (43)	ENSG00000115414
LOC387758	AI802391	DOWN	Vecchi (43)	ENSG00000176971
SGNE1	NM_003020	DOWN	Vecchi (43)	ENSG00000166922
STEAP	NM_012449	DOWN	Vecchi (43)	ENSG00000164647
RBP7	AI733027	DOWN	Vecchi (43)	ENSG00000162444
FN1	AF130095	DOWN	Vecchi (43)	ENSG00000115414
MMP2	NM_004530	DOWN	Vecchi (43)	ENSG00000087245
FN1	AK026737	DOWN	Vecchi (43)	ENSG00000115414
DCN	AF138303	DOWN	Vecchi (43)	ENSG00000011465
DCN	AF138302	DOWN	Vecchi (43)	ENSG00000011465
PRSS11	NM_002775	DOWN	Vecchi (43)	ENSG00000166033
SPON1	AI885290	DOWN	Vecchi (43)	ENSG00000262655
ETV1	BE881590	DOWN	Vecchi (43)	ENSG00000006468
GULP1	NM_016315	DOWN	Vecchi (43)	ENSG00000144366
MGP	NM_000900	DOWN	Vecchi (43)	ENSG00000111341
FMO1	NM_002021	DOWN	Vecchi (43)	ENSG00000010932
CSPG2	BF590263	DOWN	Vecchi (43)	ENSG00000038427
MFAP5	U37283	DOWN	Vecchi (43)	ENSG00000197614
CTHRC1	AA584310	DOWN	Vecchi (43)	ENSG00000164932
LRRC17	NM_005824	DOWN	Vecchi (43)	ENSG00000128606
GPM6B	AI419030	DOWN	Vecchi (43)	ENSG00000046653
CHI3L1	M80927	DOWN	Vecchi (43)	ENSG00000133048
OMD	NM_005014	DOWN	Vecchi (43)	ENSG00000127083
CTSK	NM_000396	DOWN	Vecchi (43)	ENSG00000143387
EDIL3	AA053711	DOWN	Vecchi (43)	ENSG00000164176
CPA3	NM_001870	DOWN	Vecchi (43)	ENSG00000163751
DIO2	NM_013989	DOWN	Vecchi (43)	ENSG00000211448
PTN	AL565812	DOWN	Vecchi (43)	ENSG00000105894
IRX2	AI928035	DOWN	Vecchi (43)	ENSG00000170561
TNFAIP6	AW188198	DOWN	Vecchi (43)	ENSG00000123610
WNT2	NM_003391	DOWN	Vecchi (43)	ENSG00000105989
VSNL1	AF039555	DOWN	Vecchi (43)	ENSG00000163032
FST	NM_013409	DOWN	Vecchi (43)	ENSG00000134363
COL1A2	AA628535	DOWN	Vecchi (43)	ENSG00000164692
MFAP5	AW665892	DOWN	Vecchi (43)	ENSG00000197614
LOC83690	AF142573	DOWN	Vecchi (43)	ENSG00000121005
TNFAIP6	NM_007115	DOWN	Vecchi (43)	ENSG00000123610
TNN	BF432086	DOWN	Vecchi (43)	ENSG00000120332
DIO2	U53506	DOWN	Vecchi (43)	ENSG00000211448
C20orf82	AI816793	DOWN	Vecchi (43)	ENSG00000101230
LOC83468	W63754	DOWN	Vecchi (43)	ENSG00000120820
MAP3K4	AI633559	DOWN	Vecchi (43)	ENSG00000085511
PIP	NM_002652	DOWN	Vecchi (43)	ENSG00000159763
ALDH1A3	NM_000693	DOWN	Vecchi (43)	ENSG00000184254
FBLN1	Z95331	DOWN	Vecchi (43)	ENSG00000077942
COL3A1	AU146808	DOWN	Vecchi (43)	ENSG00000168542
ASPN	NM_017680	DOWN	Vecchi (43)	ENSG00000106819
PTN	BC005916	DOWN	Vecchi (43)	ENSG00000105894
ITGBL1	AL359052	DOWN	Vecchi (43)	ENSG00000198542
PDGFRL	NM_006207	DOWN	Vecchi (43)	ENSG00000104213
ODZ2	AB032953	DOWN	Vecchi (43)	ENSG00000145934
APOD	NM_001647	DOWN	Vecchi (43)	ENSG00000189058
POSTN	D13665	DOWN	Vecchi (43)	ENSG00000133110
DIO2	AI038059	DOWN	Vecchi (43)	ENSG00000211448
MMP7	NM_002423	DOWN	Vecchi (43)	ENSG00000137673
S100A2	NM_005978	DOWN	Vecchi (43)	ENSG00000196754
KRT6B	AI831452	DOWN	Vecchi (43)	ENSG00000185479
FIBL-6	BF446673	DOWN	Vecchi (43)	ENSG00000143341
FAP	U76833	DOWN	Vecchi (43)	ENSG00000078098
OGN	AV700059	DOWN	Vecchi (43)	ENSG00000106809
ITGBL1	NM_004791	DOWN	Vecchi (43)	ENSG00000198542
MME	AI433463	DOWN	Vecchi (43)	ENSG00000196549
LTF	NM_002343	DOWN	Vecchi (43)	ENSG00000012223
SERPINB5	NM_002639	DOWN	Vecchi (43)	ENSG00000206075
TAC1	NM_003182	DOWN	Vecchi (43)	ENSG00000006128
CHI3L1	M80927	DOWN	Vecchi (43)	ENSG00000133048
COL11A1	NM_001854	DOWN	Vecchi (43)	ENSG00000060718
FST	BF438173	DOWN	Vecchi (43)	ENSG00000134363
SFRP2	AF311912	DOWN	Vecchi (43)	ENSG00000145423
DST	NM_001723	DOWN	Vecchi (43)	ENSG00000151914
COL11A1	J04177	DOWN	Vecchi (43)	ENSG00000060718
MMP3	NM_002422	DOWN	Vecchi (43)	ENSG00000149968
F2RL2	AI378647	DOWN	Vecchi (43)	ENSG00000164220
SFRP2	AW003584	DOWN	Vecchi (43)	ENSG00000145423
KRT14	BC002690	DOWN	Vecchi (43)	ENSG00000186847
