# Supplementary material for: Molecular patterns of cancer colonisation in lymph nodes of breast cancer patients
Source: Breast Cancer Res. 2018 Nov 20;20:143. doi: 10.1186/s13058-018-1070-3 (PMC6247766; doi:10.1186/s13058-018-1070-3)
Supplement: Supplementary file 2 — Table S2. Genes found to be differentially expressed in multiple studies included in scenario 1 (involved LN versus primary breast tumour). (PDF 18 kb) [file 13058_2018_1070_MOESM2_ESM.pdf]

HGNC gene symbol	ensembl gene id	Number of studies	Up or down reguated
COL11A1	ENSG00000060718	4	DOWN, DOWN, DOWN, DOWN
ASPN	ENSG00000106819	4	DOWN, DOWN, DOWN, DOWN
C7	ENSG00000112936	4	UP, UP, UP, UP
POSTN, OSF-2	ENSG00000133110	4	DOWN, DOWN, DOWN, DOWN
LUM	ENSG00000139329	4	UP, DOWN, DOWN, DOWN
MRC2	ENSG00000011028	3	DOWN, DOWN, DOWN
SNAI2	ENSG00000019549	3	DOWN, DOWN, DOWN
EPHA3	ENSG00000044524	3	DOWN, UP, UP
FBLN1	ENSG00000077942	3	DOWN, DOWN, DOWN
MMP2	ENSG00000087245	3	DOWN, DOWN, DOWN
PDGFRL	ENSG00000104213	3	DOWN, DOWN, DOWN
OGN	ENSG00000106809	3	DOWN, DOWN, DOWN
SPARC	ENSG00000113140	3	DOWN, DOWN, DOWN
TNFAIP6	ENSG00000123610	3	DOWN, DOWN, DOWN
F13A1	ENSG00000124491	3	DOWN, DOWN, DOWN
GPNMB	ENSG00000136235	3	UP, DOWN, DOWN
MMP7	ENSG00000137673	3	DOWN, DOWN, DOWN
CTSK	ENSG00000143387	3	DOWN, DOWN, DOWN
STEAP	ENSG00000164647	3	DOWN, DOWN, DOWN
COL1A2	ENSG00000164692	3	DOWN, DOWN, DOWN
HTRA1, PRSS11	ENSG00000166033	3	UP, DOWN, DOWN
GAS1	ENSG00000180447	3	DOWN, DOWN, DOWN
KRT14	ENSG00000186847	3	DOWN, DOWN, DOWN
MME	ENSG00000196549	3	DOWN, DOWN, DOWN
ITGBL1	ENSG00000198542	3	DOWN, DOWN, DOWN
COL5A2	ENSG00000204262	3	DOWN, DOWN, DOWN
DIO2	ENSG00000211448	3	DOWN, DOWN, DOWN
SPON1	ENSG00000262655	3	DOWN, DOWN, DOWN
TAC1	ENSG00000006128	2	DOWN, DOWN
FMO1	ENSG00000010932	2	DOWN, DOWN
DCN	ENSG00000011465	2	DOWN, DOWN
BIRC3	ENSG00000023445	2	UP, UP
CSPG2	ENSG00000038427	2	DOWN, DOWN
PTPRC	ENSG00000081237	2	DOWN, UP
LGALS1	ENSG00000100097	2	UP, DOWN
TIMP3	ENSG00000100234	2	UP, DOWN
MXRA5, DKFZp564I1922	ENSG00000101825	2	DOWN, DOWN
PLS3	ENSG00000102024	2	UP, DOWN
TIMP1	ENSG00000102265	2	UP, UP
PTN	ENSG00000105894	2	DOWN, DOWN
WNT2	ENSG00000105989	2	DOWN, DOWN
ART4	ENSG00000111339	2	UP, UP
FN1	ENSG00000115414	2	DOWN, DOWN
SDC1	ENSG00000115884	2	UP, DOWN
SFRS11	ENSG00000116754	2	DOWN, DOWN
CD48	ENSG00000117091	2	DOWN, UP
CR2	ENSG00000117322	2	DOWN, UP
TNFAIP3	ENSG00000118503	2	UP, UP
TNN	ENSG00000120332	2	DOWN, DOWN
OMD	ENSG00000127083	2	DOWN, DOWN
KRT17	ENSG00000128422	2	DOWN, DOWN
CALU	ENSG00000128595	2	DOWN, DOWN
COL5A1	ENSG00000130635	2	DOWN, DOWN
CHI3L1	ENSG00000133048	2	DOWN, DOWN
FST	ENSG00000134363	2	DOWN, DOWN
GRP	ENSG00000134443	2	DOWN, DOWN
MMP13	ENSG00000137745	2	DOWN, DOWN
COL6A1	ENSG00000142156	2	DOWN, DOWN
GULP1	ENSG00000144366	2	DOWN, DOWN
SFRP2	ENSG00000145423	2	DOWN, DOWN
IGFBP3	ENSG00000146674	2	DOWN, DOWN
MMP3	ENSG00000149968	2	DOWN, DOWN
DST	ENSG00000151914	2	DOWN, DOWN
ZFP36L2	ENSG00000152518	2	UP, UP
SPARCL1	ENSG00000152583	2	DOWN, DOWN
MBNL1	ENSG00000152601	2	UP, UP
MS4A1	ENSG00000156738	2	UP, UP
SPON2	ENSG00000159674	2	DOWN, DOWN
VSNL1	ENSG00000163032	2	UP, DOWN
CPA3	ENSG00000163751	2	DOWN, DOWN
F2RL2	ENSG00000164220	2	DOWN, DOWN
CTHRC1	ENSG00000164932	2	DOWN, DOWN
FBN1	ENSG00000166147	2	DOWN, DOWN
MMP10	ENSG00000166670	2	DOWN, DOWN
SCG5, SGNE1	ENSG00000166922	2	DOWN, DOWN
GREM1	ENSG00000166923	2	DOWN, DOWN
COL3A1	ENSG00000168542	2	DOWN, DOWN
CDW52	ENSG00000169442	2	UP, UP
LRRC15	ENSG00000172061	2	DOWN, DOWN
CCL19	ENSG00000172724	2	UP, UP
KRT6B	ENSG00000185479	2	DOWN, DOWN
SELL	ENSG00000188404	2	UP, UP
APOD	ENSG00000189058	2	DOWN, DOWN
MMP1	ENSG00000196611	2	DOWN, DOWN
S100A2	ENSG00000196754	2	DOWN, DOWN
MFAP5	ENSG00000197614	2	DOWN, DOWN
LTB	ENSG00000227507	2	UP, UP
TXNIP	ENSG00000265972	2	DOWN, DOWN
