# Supplementary material for: Molecular patterns of cancer colonisation in lymph nodes of breast cancer patients
Source: Breast Cancer Res. 2018 Nov 20;20:143. doi: 10.1186/s13058-018-1070-3 (PMC6247766; doi:10.1186/s13058-018-1070-3)
Supplement: Supplementary file 3 — Table S3. Fully compiled gene list across all scenarios. (PDF 75 kb) [file 13058_2018_1070_MOESM3_ESM.pdf]

Ensembl ID	Gene Name	Scenario 1	Scenario 2	Scenario 3	Scenario 4	Scenario 5	Scenario 6
ENSG00000189058	APOD	D	D				
ENSG00000186081	KRT5	D					
ENSG00000167244	IGF2	D					
ENSG00000137699	TRIM29	D					
ENSG00000169429	CXCL8	D					
ENSG00000077942	FBLN1	D					
ENSG00000265190	ANXA8	D					
ENSG00000124491	F13A1	D					
ENSG00000196549	MME	D	D				
ENSG00000211448	DIO2	D					
ENSG00000089472	HEPH	D					
ENSG00000164120	HPGD	D					
ENSG00000144366	GULP1	D					
ENSG00000196754	S100A2	D					
ENSG00000151914	DST	D					
ENSG00000180447	GAS1	D					
ENSG00000196611	MMP1	D					
ENSG00000155659	VSIG4	D					
ENSG00000094755	GABRP	D					
ENSG00000128422	KRT17	D					
ENSG00000163751	CPA3	D					
ENSG00000166670	MMP10	D					
ENSG00000127083	OMD	D	D				
ENSG00000083782	EPYC	D					
ENSG00000164220	F2RL2	D	D				
ENSG00000184408	KCND2	D					
ENSG00000117586	TNFSF4	D					
ENSG00000011465	DCN	D	D				
ENSG00000133048	CHI3L1	D					
ENSG00000105894	PTN	D	D				
ENSG00000178363	CALML3	D					
ENSG00000146674	IGFBP3	D					
ENSG00000019549	SNAI2	D					
ENSG00000153707	PTPRD	D					
ENSG00000106236	NPTX2	D					
ENSG00000185479	KRT6B	D					
ENSG00000101134	DOK5	D					
ENSG00000166923	GREM1	D					
ENSG00000198759	EGFL6	D					
ENSG00000162493	PDPN	D					
ENSG00000044524	EPHA3	U					
ENSG00000135914	HTR2B	D					
ENSG00000158477	CD1A	D					
ENSG00000155011	DKK2	D					
ENSG00000204580	DDR1	D					
ENSG00000271503	CCL5	D					
ENSG00000182580	EPHB3	D					
ENSG00000255974	CYP2A6	D					U
ENSG00000157227	MMP14	D					
ENSG00000174231	PRPF8	D					
ENSG00000100353	EIF3D	D					
ENSG00000117410	ATP6V0B	D					
ENSG00000147065	MSN	D					
ENSG00000096696	DSP	D					
ENSG00000142949	PTPRF	D					
ENSG00000163191	S100A11	D					
ENSG00000113140	SPARC	D					
ENSG00000100219	XBP1	D					
ENSG00000115306	SPTBN1	D					
ENSG00000116754	SRSF11	D					
ENSG00000136240	KDELR2	D					
ENSG00000113558	SKP1	D					
ENSG00000128595	CALU	D					
ENSG00000144746	ARL6IP5	D					
ENSG00000044115	CTNNA1	D					
ENSG00000117984	CTSD	D					
ENSG00000115758	ODC1	D					
ENSG00000204389	HSPA1A	D					
ENSG00000083444	PLOD1	D					
ENSG00000099194	SCD	D					
ENSG00000116133	DHCR24	D					
ENSG00000166165	CKB	D					
ENSG00000155367	PPM1J	D					
ENSG00000004478	FKBP4	D					
ENSG00000129116	PALLD	D					
ENSG00000198408	MGEA5	D					
ENSG00000204592	HLA-E	D					
ENSG00000147526	TACC1	D					
ENSG00000133639	BTG1	D					
ENSG00000108679	LGALS3BP	D					
ENSG00000230989	HSBP1	D					
ENSG00000118971	CCND2	D					
ENSG00000204634	TBC1D8	D					
ENSG00000099204	ABLIM1	D					
ENSG00000197043	ANXA6	D					
ENSG00000136026	CKAP4	D					
ENSG00000010278	CD9	D					
ENSG00000265972	TXNIP	D					
ENSG00000173801	JUP	D					
ENSG00000125868	DSTN	D					

ENSG00000111716	LDHB	U					
ENSG00000148700	ADD3	U					
ENSG00000085733	CTTN	U					
ENSG00000100097	LGALS1	B					
ENSG00000137801	THBS1	U					
ENSG00000082781	ITGB5	U				U	
ENSG00000039068	CDH1	U					
ENSG00000223865	HLA-DPB1	U					
ENSG00000136235	GPNMB	D					
ENSG00000100234	TIMP3	B					
ENSG00000152601	MBNL1	U					
ENSG00000166033	HTRA1	D					
ENSG00000215301	DDX3X	U					
ENSG00000102024	PLS3	B					
ENSG00000175029	CTBP2	U					
ENSG00000019995	ZRANB1	U					
ENSG00000143153	ATP1B1	U					
ENSG00000008282	SYPL1	U					
ENSG00000182492	BGN	U					
ENSG00000115884	SDC1	B					
ENSG00000111348	ARHGDIB	U					
ENSG00000142871	CYR61	U		U			
ENSG00000114978	MOB1A	U					
ENSG00000136938	ANP32B	U					
ENSG00000134986	NREP	U					
ENSG00000134531	EMP1	U					
ENSG00000157557	ETS2	U					
ENSG00000171617	ENC1	U					
ENSG00000109062	SLC9A3R1	U					
ENSG00000152518	ZFP36L2	U					
ENSG00000154277	UCHL1	U					
ENSG00000173020	GRK2	U					
ENSG00000143198	MGST3	U					
ENSG00000124766	SOX4	U					
ENSG00000189143	CLDN4	U					
ENSG00000113657	DPYSL3	U					
ENSG00000163359	COL6A3	U					
ENSG00000181019	NQO1	U					
ENSG00000109861	CTSC	U					
ENSG00000137509	PRCP	U					
ENSG00000091136	LAMB1	U					
ENSG00000141753	IGFBP4	U					
ENSG00000163435	ELF3	U					
ENSG00000132383	RPA1	U					
ENSG00000022267	FHL1	U	D				
ENSG00000079332	SAR1A	U					
ENSG00000117139	KDM5B	U					
ENSG00000140263	SORD	U					
ENSG00000075618	FSCN1	U					
ENSG00000239672	NME1	U					
ENSG00000083857	FAT1	U					
ENSG00000182718	ANXA2	U					
ENSG00000111057	KRT18	U					
ENSG00000064666	CNN2	U					
ENSG00000158747	NBL1	U					
ENSG00000137331	IER3	U					
ENSG00000162437	RAVER2	U					
ENSG00000171345	KRT19	U			U		
ENSG00000123983	ACSL3	U					
ENSG00000102265	TIMP1	U					
ENSG00000152661	GJA1	U					
ENSG00000151208	DLG5	U					
ENSG00000076554	TPD52	U					
ENSG00000112576	CCND3	U					
ENSG00000197586	ENTPD6	U					
ENSG00000131462	TUBG1	U					
ENSG00000079819	EPB41L2	U					
ENSG00000162511	LAPTM5	U					
ENSG00000139329	LUM	D					
ENSG00000151239	TWF1	U					
ENSG00000164919	COX6C	U					
ENSG00000100297	MCM5	U					
ENSG00000134291	TMEM106C	U					
ENSG00000110711	AIP	U					
ENSG00000172893	DHCR7	U					
ENSG00000106624	AEBP1	U					
ENSG00000143815	LBR	U					
ENSG00000138119	MYOF	U					
ENSG00000131370	SH3BP5	U					
ENSG00000196683	TOMM7	U					
ENSG00000134375	TIMM17A	U					
ENSG00000013561	RNF14	U					
ENSG00000143653	SCCPDH	U					
ENSG00000119888	EPCAM	U			U		
ENSG00000106211	HSPB1	U					
ENSG00000115380	EFEMP1	U					
ENSG00000176171	BNIP3	U					
ENSG00000145423	SFRP2	D	D				
ENSG00000060718	COL11A1	D					
ENSG00000104213	PDGFRL	D					
ENSG00000087245	MMP2	D					

ENSG00000143387	CTSK	D					
ENSG00000137673	MMP7	D					
ENSG00000106819	ASPN	D					
ENSG00000198542	ITGBL1	D					
ENSG00000133110	POSTN	D					
ENSG00000142156	COL6A1	D					
ENSG00000010932	FMO1	D	D				
ENSG00000204262	COL5A2	D					
ENSG00000170801	HTRA3	D					
ENSG00000152377	SPOCK1	D					
ENSG00000262655	SPON1	D					
ENSG00000138615	CILP	D					
ENSG00000081237	PTPRC	U					
ENSG00000106809	OGN	D	D				
ENSG00000143369	ECM1	D					
ENSG00000164647	STEAP1	D					
ENSG00000115414	FN1	D				U	
ENSG00000123610	TNFAIP6	D					
ENSG00000164692	COL1A2	D				U	
ENSG00000168542	COL3A1	D				U	
ENSG00000166147	FBN1	D					
ENSG00000108700	CCL8	D					
ENSG00000038427	VCAN	D					
ENSG00000124813	RUNX2	D					
ENSG00000134443	GRP	D					D
ENSG00000106333	PCOLCE	D					
ENSG00000119699	TGFB3	D					
ENSG00000130635	COL5A1	D					
ENSG00000093009	CDC45	D					
ENSG00000186847	KRT14	D					
ENSG00000164932	CTHRC1	D					
ENSG00000131378	RFTN1	D					
ENSG00000011028	MRC2	D					
ENSG00000249992	TMEM158	D					
ENSG00000102879	CORO1A	D					
ENSG00000203747	FCGR3A	D					
ENSG00000150907	FOXO1	D					
ENSG00000117322	CR2	B					
ENSG00000117091	CD48	B					
ENSG00000004799	PDK4	D					
ENSG00000103222	ABCC1	D					
ENSG00000122862	SRGN	D					
ENSG00000276886	GREM1	D					
ENSG00000139053	PDE6H	U					
ENSG00000156738	MS4A1	U					
ENSG00000112936	C7	U					
ENSG00000227507	LTB	U					
ENSG00000188404	SELL	U					
ENSG00000090104	RGS1	U					
ENSG00000132185	FCRLA	U					D
ENSG00000023445	BIRC3	U					
ENSG00000118503	TNFAIP3	U					
ENSG00000158050	DUSP2	U					
ENSG00000244734	HBB	U					
ENSG00000100721	TCL1A	U					
ENSG00000127528	KLF2	U					
ENSG00000169508	GPR183	U					
ENSG00000171346	KRT15	U					U
ENSG00000172724	CCL19	U					
ENSG00000277586	NEFL	U					
ENSG00000162896	PIGR	U	D				
ENSG00000141293	SKAP1	U					
ENSG00000125952	MAX	U					
ENSG00000128609	NDUFA5	U					
ENSG00000119688	ABCD4	U					
ENSG00000142627	EPHA2	U					
ENSG00000175567	UCP2	U					
ENSG00000120129	DUSP1	U		U			
ENSG00000108947	EFNB3	U					
ENSG00000185201	IFITM2	D					
ENSG00000142089	IFITM3	D					
ENSG00000155760	FZD7	D					
ENSG00000035862	TIMP2	D					
ENSG00000172757	CFL1	D					
ENSG00000052802	MSMO1	D					
ENSG00000142875	PRKACB	D					
ENSG00000150093	ITGB1	D					
ENSG00000117118	SDHB	D					
ENSG00000137745	MMP13	D					
ENSG00000196177	ACADSB	D					
ENSG00000149968	MMP3	D					U
ENSG00000185745	IFIT1	D					
ENSG00000182985	CADM1	D					
ENSG00000101955	SRPX	D	D				
ENSG00000152583	SPARCL1	D	D				
ENSG00000141526	SLC16A3	D					
ENSG00000111319	SCNN1A	D					U
ENSG00000168461	RAB31	D					
ENSG00000074590	NUAK1	D					
ENSG00000170525	PFKFB3	D					
ENSG00000168209	DDIT4	D					

ENSG00000172638	EFEMP2	D					
ENSG00000099860	GADD45B	D					
ENSG00000169241	SLC50A1	D					
ENSG00000067064	IDI1	D					
ENSG00000125810	CD93	D					
ENSG00000085063	CD59	D					
ENSG00000169504	CLIC4	D					
ENSG00000137814	HAUS2	D					
ENSG00000072682	P4HA2	D					
ENSG00000109046	WSB1	D					
ENSG00000049860	HEXB	D					
ENSG00000087076	HSD17B14	D					
ENSG00000156234	CXCL13	U					
ENSG00000170476	MZB1	U					
ENSG00000163032	VSNL1	B					
ENSG00000107317	PTGDS	U					
ENSG00000007516	BAIAP3	U					
ENSG00000171992	SYNPO	U					
ENSG00000175003	SLC22A1	U					
ENSG00000169442	CD52	U					
ENSG00000186575	NF2	U					
ENSG00000126457	PRMT1	U					
ENSG00000137203	TFAP2A	U			U		
ENSG00000160321	ZNF208	U					
ENSG00000147168	IL2RG	U					
ENSG00000133124	IRS4	U					
ENSG00000143786	CNIH3	D					
ENSG00000134363	FST	D					
ENSG00000139330	KERA	D					
ENSG00000172061	LRRC15	D					
ENSG00000156103	MMP16	D	D				
ENSG00000101825	MXRA5	D					
ENSG00000197614	MFAP5	D					
ENSG00000157168	NRG1	D					
ENSG00000138207	RBP4	D					
ENSG00000166922	SCG5	D					
ENSG00000159674	SPON2	D					
ENSG00000006128	TAC1	D					
ENSG00000120332	TNN	D					
ENSG00000184937	WT1	D					
ENSG00000105989	WNT2	D					
ENSG00000111339	ART4	U					
ENSG00000150636	CCDC102B	U					
ENSG00000137077	CCL21	U					
ENSG00000007312	CD79B	U					
ENSG00000081052	COL4A4	U					
ENSG00000163687	DNASE1L3	U					
ENSG00000103241	FOXF1	U					
ENSG00000176165	FOXG1	U					
ENSG00000083454	P2RX5	U					
ENSG00000153064	BANK1	U					
ENSG00000147606	SLC26A7	U					
ENSG00000196092	PAX5	U					D
ENSG00000181617	FDCSP	U					D
ENSG00000163534	FCRL1	U					
ENSG00000009790	TRAF3IP3	U					
ENSG00000110777	POU2AF1	U					
ENSG00000119866	BCL11A	U					D
ENSG00000125245	GPR18	U					
ENSG00000035720	STAP1	U					
ENSG00000163519	TRAT1	U					
ENSG00000173698	ADGRG2	U					
ENSG00000118308	LRMP	U					
ENSG00000271856	LINC01215	U					
ENSG00000169031	COL4A3	U					
ENSG00000127152	BCL11B	U					
ENSG00000211899	IGHM	U					
ENSG00000162894	FCMR	U					
ENSG00000174946	GPR171	U					
ENSG00000241106	HLA-DOB	U					
ENSG00000185811	IKZF1	U					
ENSG00000091490	SEL1L3	U					
ENSG00000168685	IL7R	U					
ENSG00000115165	CYTIP	U					
ENSG00000106511	MEOX2	U					
ENSG00000164691	TAGAP	U					
ENSG00000145287	PLAC8	U					
ENSG00000160856	FCRL3	U					
ENSG00000124406	ATP8A1	U					
ENSG00000078081	LAMP3	U					
ENSG00000121966	CXCR4	U		U			
ENSG00000170542	SERPINB9	U					
ENSG00000182866	LCK	U					
ENSG00000180448	ARHGAP45	U					
ENSG00000126353	CCR7	U					
ENSG00000100985	MMP9	U					
ENSG00000100368	CSF2RB	U					
ENSG00000055163	CYFIP2	U					
ENSG00000213658	LAT	U					D
ENSG00000134602	STK26	U					
ENSG00000162692	VCAM1	U					

ENSG00000145779	TNFAIP8	U					
ENSG00000128340	RAC2	U					
ENSG00000172575	RASGRP1	U					
ENSG00000166501	PRKCB	U					D
ENSG00000167286	CD3D	U					
ENSG00000054219	LY75	U					
ENSG00000172005	MAL	U					
ENSG00000081320	STK17B	U					
ENSG00000185862	EVI2B	U					
ENSG00000196950	SLC39A10	U					
ENSG00000152270	PDE3B	U					
ENSG00000158987	RAPGEF6	U					
ENSG00000197943	PLCG2	U					
ENSG00000187239	FNBP1	U					
ENSG00000204267	TAP2	U					
ENSG00000073849	ST6GAL1	U					
ENSG00000140968	IRF8	U					
ENSG00000134954	ETS1	U					
ENSG00000047365	ARAP2	U					
ENSG00000169499	PLEKHA2	U					
ENSG00000077943	ITGA8	U					
ENSG00000100077	GRK3	U					
ENSG00000182568	SATB1	U					
ENSG00000115419	GLS	U					
ENSG00000143119	CD53	U					
ENSG00000095585	BLNK	U					
ENSG00000071564	TCF3	U					
ENSG00000275066	SYNRG	U					
ENSG00000174718	KIAA1551	U					
ENSG00000112149	CD83	U					
ENSG00000084070	SMAP2	U					
ENSG00000163545	NUAK2	U					
ENSG00000137478	FCHSD2	U					
ENSG00000104880	ARHGEF18	U					
ENSG00000146094	DOK3	U					D
ENSG00000112365	ZBTB24	U					
ENSG00000103653	CSK	U					
ENSG00000110031	LPXN	U					
ENSG00000241978	AKAP2	U					
ENSG00000073282	TP63	D					
ENSG00000135046	ANXA1	D					
ENSG00000137273	FOXF2	D					
ENSG00000140937	CDH11	D					
ENSG00000131981	LGALS3	D					
ENSG00000103196	CRISPLD2	D					
ENSG00000156804	FBXO32	D					
ENSG00000149591	TAGLN	D					
ENSG00000141568	FOXK2	D					
ENSG00000120594	PLXDC2	D					
ENSG00000107796	ACTA2	D					
ENSG00000102125	TAZ	D					
ENSG00000164949	GEM	D					
ENSG00000086991	NOX4	D					
ENSG00000166741	NNMT	D					
ENSG00000116774	OLFML3	D					
ENSG00000244731	C4A	D					
ENSG00000046653	GPM6B	D					
ENSG00000122691	TWIST1	D					
ENSG00000189184	PCDH18	D					
ENSG00000013297	CLDN11	D					
ENSG00000148848	ADAM12	D					
ENSG00000118785	SPP1	D					
ENSG00000174099	MSRB3	D					
ENSG00000108821	COL1A1	D				U	
ENSG00000186340	THBS2	D					
ENSG00000157404	KIT	D					
ENSG00000163106	HPGDS	D			D		
ENSG00000091986	CCDC80	D					
ENSG00000118523	CTGF	D		U			
ENSG00000196139	AKR1C3	D					
ENSG00000176971	FIBIN	D					
ENSG00000162444	RBP7	D					
ENSG00000006468	ETV1	D					
ENSG00000111341	MGP	D					
ENSG00000128606	LRRC17	D	D				
ENSG00000164176	EDIL3	D					
ENSG00000170561	IRX2	D					
ENSG00000121005	CRISPLD1	D					
ENSG00000101230	ISM1	D					
ENSG00000120820	GLT8D2	D					
ENSG00000085511	MAP3K4	D					
ENSG00000159763	PIP	D			U		U
ENSG00000184254	ALDH1A3	D					
ENSG00000145934	TENM2	D					
ENSG00000143341	HMCN1	D	D				
ENSG00000078098	FAP	D					
ENSG00000012223	LTF	D					
ENSG00000206075	SERPINB5	D					
ENSG00000199575	SNORD114-1		D				
ENSG00000170323	FABP4		D				
ENSG00000174697	LEP		D				

ENSG00000173467	AGR3		D				
ENSG00000120738	EGR1		D				
ENSG00000240666	MME-AS1		D				
ENSG00000113594	LIFR		D				
ENSG00000135218	CD36		D				
ENSG00000214548	MEG3		D				
ENSG00000283874	MIR3911		D				
ENSG00000196616	ADH1B		D				
ENSG00000200480	SNORD114-28		D				
ENSG00000182463	TSHZ2		D				
ENSG00000197953	AADACL2		D				
ENSG00000144642	RBMS3		D				
ENSG00000137558	PI15		D				
ENSG00000154721	JAM2		D				
ENSG00000139865	TTC6		D				
ENSG00000174059	CD34		D				
ENSG00000152580	IGSF10		D				
ENSG00000165124	SVEP1		D				
ENSG00000197776	KLHDC1		D				
ENSG00000267532	MIR497HG		D				
ENSG00000189056	RELN		D				
ENSG00000114698	PLSCR4		D				
ENSG00000163491	NEK10		D				
ENSG00000078596	ITM2A		D				
ENSG00000139211	AMIGO2		D				
ENSG00000139910	NOVA1		D				
ENSG00000266514	MIR3689F		D				
ENSG00000147257	GPC3		D				
ENSG00000016402	IL20RA		D				
ENSG00000123243	ITIH5		D				
ENSG00000136158	SPRY2		D				
ENSG00000196208	GREB1		D				U
ENSG00000164035	EMCN		D				
ENSG00000185924	RTN4RL1		D				
ENSG00000103495	MAZ		U				
ENSG00000175643	RMI2		U				
ENSG00000135750	KCNK1		U				
ENSG00000102384	CENPI		U				
ENSG00000166797	FAM96A		U				
ENSG00000135829	DHX9		U				
ENSG00000164109	MAD2L1		U				
ENSG00000102524	TNFSF13B		U				
ENSG00000184661	CDCA2		U				
ENSG00000169679	BUB1		U				
ENSG00000138346	DNA2		U				
ENSG00000170540	ARL6IP1		U				
ENSG00000138071	ACTR2		U				
ENSG00000175063	UBE2C		U				
ENSG00000136522	MRPL47		U				
ENSG00000173726	TOMM20		U				
ENSG00000102172	SMS		U				
ENSG00000115415	STAT1		U				
ENSG00000137804	NUSAP1		U				
ENSG00000121644	DESI2		U				
ENSG00000182481	KPNA2		U				
ENSG00000138160	KIF11		U				
ENSG00000146386	ABRACL		U				
ENSG00000203668	CHML		U				
ENSG00000163468	CCT3		U				
ENSG00000080986	NDC80		U				
ENSG00000011426	ANLN		U				
ENSG00000277075	HIST1H2AE		U				
ENSG00000117724	CENPF		U				
ENSG00000133136	GNG5P2		U				
ENSG00000088325	TPX2		U				
ENSG00000184270	HIST2H2AB		U				
ENSG00000277775	HIST1H3F		U				
ENSG00000034510	TMSB10		U				
ENSG00000123975	CKS2		U				
ENSG00000131747	TOP2A		U				
ENSG00000148513	ANKRD30A		D				U
ENSG00000211890	IGHA2		D				
ENSG00000184258	CDR1		D				
ENSG00000212384	SNORD113-2		D				
ENSG00000201839	SNORD114-3		D				
ENSG00000200823	SNORD114-2		D				
ENSG00000187955	COL14A1		D				
ENSG00000154262	ABCA6		D				
ENSG00000004776	HSPB6		D				
ENSG00000154175	ABI3BP		D				
ENSG00000017427	IGF1		D				
ENSG00000270641	TSIX		D				
ENSG00000107562	CXCL12		D				
ENSG00000154263	ABCA10		D				
ENSG00000201700	SNORD113-3		D				
ENSG00000272344	SNORD114-21		D				
ENSG00000141338	ABCA8		D				
ENSG00000070193	FGF10		D				
ENSG00000109472	CPE		D				
ENSG00000199390	SNORD114-7		D				
ENSG00000201557	SNORD114-15		D				

ENSG00000127920	GNG11		D				
ENSG00000196569	LAMA2		D				
ENSG00000114771	AADAC		D				
ENSG00000173432	SAA1		D				
ENSG00000200612	SNORD114-25		D				
ENSG00000264424	MYH4		D				
ENSG00000106483	SFRP4		D				
ENSG00000164124	TMEM144		D				
ENSG00000133083	DCLK1		D				
ENSG00000109686	SH3D19		D				
ENSG00000126785	RHOJ		D				
ENSG00000205364	MT1M		D				
ENSG00000130176	CNN1		D				
ENSG00000164946	FREM1		D				
ENSG00000206384	COL6A6		D				
ENSG00000134138	MEIS2		D				
ENSG00000121297	TSHZ3		D				
ENSG00000178081	ULK4P3		D				
ENSG00000136160	EDNRB		D				
ENSG00000144218	AFF3		D				
ENSG00000053328	METTL24		D				
ENSG00000169744	LDB2		D				
ENSG00000092009	CMA1		D				
ENSG00000203697	CAPN8		D				
ENSG00000164741	DLC1		D				
ENSG00000088992	TESC		D				
ENSG00000091656	ZFHX4		D				
ENSG00000197635	DPP4		D				
ENSG00000156136	DCK		U				
ENSG00000139826	ABHD13		U				
ENSG00000181163	NPM1		U				
ENSG00000147669	POLR2K		U				
ENSG00000160813	PPP1R35		U				
ENSG00000076382	SPAG5		U				
ENSG00000163808	KIF15		U				
ENSG00000204604	ZNF468		U				
ENSG00000111412	C12orf49		U				
ENSG00000024526	DEPDC1		U				
ENSG00000132463	GRSF1		U				
ENSG00000239105	RNU7-73P		U				
ENSG00000126787	DLGAP5		U				
ENSG00000154839	SKA1		U				
ENSG00000170312	CDK1		U				
ENSG00000249784	SCARNA22		U				D
ENSG00000116748	AMPD1				D		
ENSG00000104938	CLEC4M				D		
ENSG00000081041	CXCL2				D		
ENSG00000163735	CXCL5				D		
ENSG00000184500	PROS1				D		
ENSG00000136011	STAB2				D		
ENSG00000003436	TFPI				D		
ENSG00000106541	AGR2				U		
ENSG00000129354	AP1M2				U		
ENSG00000160862	AZGP1				U		
ENSG00000143320	CRABP2				U		
ENSG00000129514	FOXA1				U		U
ENSG00000089356	FXYD3				U		U
ENSG00000185499	MUC1				U		U
ENSG00000189334	S100A14				U		
ENSG00000087510	TFAP2C				U		
ENSG00000188157	AGRN					U	
ENSG00000164171	ITGA2					U	
ENSG00000144668	ITGA9					D	
ENSG00000139626	ITGB7					D	
ENSG00000005961	ITGA2B					D	
ENSG00000026508	CD44					D	
ENSG00000185245	GP1BA					D	
ENSG00000115232	ITGA4					D	
ENSG00000263597	MIR3936						U
ENSG00000284567	MIR223						U
ENSG00000200983	SNORA3A						U
ENSG00000207145	SNORA18						U
ENSG00000283513	MIR941-3						U
ENSG00000207088	SNORA7B						U
ENSG00000238363	SNORA13						U
ENSG00000206612	SNORA2A						U
ENSG00000251898	SCARNA11						U
ENSG00000265810	MIR3907						U
ENSG00000239183	SNORA84						U
ENSG00000197838	CYP2A13						U
ENSG00000202363	SNORA62						U
ENSG00000233101	HOXB-AS3						U
ENSG00000163041	H3F3A						U
ENSG00000214407	LINC02085						U
ENSG00000162989	KCNJ3						U
ENSG00000205361	MT1DP						U
ENSG00000179676	LINC00305						U
ENSG00000126890	CTAG2						U
ENSG00000171446	KRT27						U
ENSG00000212899	KRTAP3-3						U
ENSG00000186910	SERPINA11						U

ENSG00000183644	C11orf88						U
ENSG00000204511	MCCD1						U
ENSG00000274618	HIST1H4F						U
ENSG00000136698	CFC1						U
ENSG00000249020	SNORA58						U
ENSG00000243543	WFDC6						U
ENSG00000124490	CRISP2						U
ENSG00000158301	GPRASP2						U
ENSG00000181359	HSP90AA6P						U
ENSG00000196800	SPINK14						U
ENSG00000276430	FAM25C						U
ENSG00000142025	DMRTC2						U
ENSG00000213931	HBE1						U
ENSG00000118245	TNP1						U
ENSG00000224689	ZNF812P						U
ENSG00000155622	XAGE2						U
ENSG00000186393	KRT26						U
ENSG00000011052	NME1-NME2						U
ENSG00000125787	GNRH2						U
ENSG00000108684	ASIC2						U
ENSG00000147689	FAM83A						U
ENSG00000152093	CFC1B						U
ENSG00000212283	SNORD89						D
ENSG00000207635	MIR499A						D
ENSG00000271798	SNORA51						D
ENSG00000210825	SNORA40						D
ENSG00000207493	SNORA46						D
ENSG00000252906	SCARNA3						D
ENSG00000207051	SNORA27						D
ENSG00000206799	SNORA32						D
ENSG00000163586	FABP1						D
ENSG00000216141	MIR941-2						D
ENSG00000216195	MIR941-4						D
ENSG00000206834	SNORA1						D
ENSG00000206633	SNORA80B						D
ENSG00000200816	SNORA38						D
ENSG00000207392	SNORA20						D
ENSG00000238835	SCARNA18						D
ENSG00000239149	SNORA59A						D
ENSG00000266079	SNORA59B						D
ENSG00000281910	SNORA50A						D
ENSG00000207313	SNORA2B						D
ENSG00000221491	SNORA2C						D
ENSG00000186146	DEFB131A						D
ENSG00000264229	RNU4ATAC						D
ENSG00000188729	OSTN						D
ENSG00000206760	SNORA6						D
ENSG00000164266	SPINK1						D
ENSG00000206634	SNORA22						D
ENSG00000243955	GSTA1						D
ENSG00000280498	SNORA16A						D
ENSG00000199266	SNORA60						D
ENSG00000235408	SNORA71B						D
ENSG00000016490	CLCA1						D
ENSG00000205649	HTN3						D
ENSG00000201772	SNORA5C						D
ENSG00000163202	LCE3D						D
ENSG00000169605	GKN1						D
ENSG00000228962	HCG23						D
ENSG00000206034	DEFB109B						D
ENSG00000165828	PRAP1						D
ENSG00000169469	SPRR1B						D
ENSG00000236552	RPL13AP5						D
ENSG00000171180	OR2M4						D
ENSG00000154646	TMPRSS15						D
ENSG00000173237	C11orf86						D
ENSG00000151418	ATP6V1G3						D
ENSG00000176009	ASCL3						D
ENSG00000167117	LINC00483						D
ENSG00000197745	SCGB1D4						D
ENSG00000254870	ATP6V1G2-DDX39B						D
ENSG00000164816	DEFA5						D
ENSG00000126550	HTN1						D
ENSG00000166869	CHP2						D
ENSG00000240108	NCOR1P1						D
ENSG00000171201	SMR3B						D
ENSG00000266200	PNLIPRP2						D
ENSG00000069482	GAL						D
ENSG00000137860	SLC28A2						D
ENSG00000118972	FGF23						D
ENSG00000129824	RPS4Y1						D
ENSG00000275126	HIST1H4L						D
ENSG00000231298	LINC00704						D
ENSG00000113430	IRX4						D
ENSG00000090402	SI						D
ENSG00000139515	PDX1						D
ENSG00000165863	C10orf82						D
ENSG00000057149	SERPINB3						D
ENSG00000169474	SPRR1A						D
ENSG00000255986	MT1JP						D
ENSG00000168333	PPDPFL						D

ENSG00000182793	GSTA5						D
ENSG00000123576	ESX1						D
ENSG00000167165	UGT1A6						D
ENSG00000136352	NKX2-1						D
ENSG00000259527	LINC00052						D
ENSG00000144820	ADGRG7						D
ENSG00000106384	MOGAT3						D
ENSG00000180066	C10orf91						D
ENSG00000171136	RLN3						D
ENSG00000277858	H2AFB2						D
ENSG00000164822	DEFA6						D
ENSG00000238205	MPC1L						D
ENSG00000082929	LINC01587						D
ENSG00000175868	CALCB						D
ENSG00000176840	MIR7-3HG						D
ENSG00000196475	GK2						D
ENSG00000179796	LRRC3B						D
ENSG00000274749	KRTAP7-1						D
ENSG00000179751	SYCN						D
ENSG00000276998	REXO1L2P						D
ENSG00000254636	ARMS2						D
ENSG00000167531	LALBA						D
ENSG00000105205	CLC						D
ENSG00000146678	IGFBP1						D
ENSG00000171557	FGG						D
ENSG00000182083	OR6B2						D
ENSG00000124469	CEACAM8						D
ENSG00000227234	SPANXB1						D
ENSG00000171431	KRT20						D
ENSG00000170477	KRT4						D
ENSG00000169575	VPREB1						D
ENSG00000114113	RBP2						D
ENSG00000136872	ALDOB						D
ENSG00000101197	BIRC7						D
ENSG00000130720	FIBCD1						D
ENSG00000173702	MUC13						D
ENSG00000162897	FCAMR						D
ENSG00000100867	DHRS2						D
ENSG00000188000	OR7D2						D
ENSG00000249948	GBA3						D
ENSG00000169903	TM4SF4						D
ENSG00000100362	PVALB						D
ENSG00000026559	KCNG1						D
ENSG00000254415	SIGLEC14						D
ENSG00000134193	REG4						D
ENSG00000104921	FCER2						D
ENSG00000170054	SERPINA9						D
ENSG00000132677	RHBG						D
ENSG00000159958	TNFRSF13C						D
ENSG00000166736	HTR3A						D
ENSG00000142449	FBN3						D
ENSG00000234965	SHISA8						D
ENSG00000173404	INSM1						D
ENSG00000167483	FAM129C						D
ENSG00000169994	MYO7B						D
ENSG00000188536	HBA2						D
ENSG00000183691	NOG						D
ENSG00000170486	KRT72						D
ENSG00000137101	CD72						D
ENSG00000177455	CD19						D
ENSG00000165178	NCF1C						D
ENSG00000104903	LYL1						D
ENSG00000012124	CD22						D
ENSG00000122224	LY9						D
ENSG00000160867	FGFR4						D
ENSG00000007129	CEACAM21						D
ENSG00000235621	LINC00494						D
ENSG00000198796	ALPK2						D
ENSG00000213199	ASIC3						D
ENSG00000260335							D
ENSG00000128218	VPREB3						D
ENSG00000138623	SEMA7A						D
ENSG00000173200	PARP15						D
ENSG00000095794	CREM						D
ENSG00000136573	BLK						D
ENSG00000204165	CXorf65						D
ENSG00000123612	ACVR1C						D
ENSG00000142512	SIGLEC10						D
ENSG00000005471	ABCB4						D
ENSG00000140379	BCL2A1						D
ENSG00000187243	MAGED4B						D
ENSG00000124334	IL9R						D
ENSG00000079263	SP140						D
ENSG00000107736	CDH23						D
ENSG00000076770	MBNL3						D
ENSG00000168237	GLYCTK						D
ENSG00000161405	IKZF3						D
ENSG00000176884	GRIN1						D
ENSG00000089012	SIRPG						D
ENSG00000166405	RIC3						D
ENSG00000103227	LMF1						D

ENSG00000130844	ZNF331						D
ENSG00000188681	TEKT4P2						D
ENSG00000131196	NFATC1						D
ENSG00000182378	PLCXD1						D
ENSG00000029534	ANK1						D
ENSG00000181722	ZBTB20						D
ENSG00000087085	ACHE						D
ENSG00000133466	C1QTNF6						D
ENSG00000090554	FLT3LG						D
ENSG00000230487	PSMG3-AS1						D
ENSG00000128604	IRF5						D
ENSG00000164620	RELL2						D
ENSG00000163239	TDRD10						D
ENSG00000239382	ALKBH6						D
ENSG00000113580	NR3C1						D
ENSG00000135299	ANKRD6						D
ENSG00000120913	PDLIM2						D
ENSG00000120539	MASTL						D
ENSG00000163564	PYHIN1						D
ENSG00000186073	C15orf41						D
ENSG00000065882	TBC1D1						D
ENSG00000133624	ZNF767P						D
ENSG00000148019	CEP78						D
ENSG00000162946	DISC1						D
ENSG00000176102	CSTF3						D
ENSG00000134256	CD101						D
ENSG00000119314	PTBP3						D
ENSG00000167757	KLK11						U
ENSG00000161055	SCGB3A1						U
ENSG00000166509	CLEC3A						U
ENSG00000129451	KLK10						U
ENSG00000186474	KLK12						U
ENSG00000167759	KLK13						U
ENSG00000198077	CYP2A7						U
ENSG00000171102	OBP2B						U
ENSG00000166006	KCNC2						U
ENSG00000153002	CPB1						U
ENSG00000089199	CHGB						U
ENSG00000205002	AARD						U
ENSG00000198650	TAT						U
ENSG00000196136	SERPINA3						U
ENSG00000169297	NR0B1						U
ENSG00000122584	NXPH1						U
ENSG00000006611	USH1C						U
ENSG00000078898	BPIFB2						U
ENSG00000138696	BMPR1B						U
ENSG00000120251	GRIA2						U
ENSG00000159212	CLIC6						U
ENSG00000108244	KRT23						U
ENSG00000119938	PPP1R3C						U
ENSG00000172551	MUCL1						U
ENSG00000178372	CALML5						U
ENSG00000136457	CHAD						U
ENSG00000039139	DNAH5						U
ENSG00000243064	ABCC13						U
ENSG00000178538	CA8						U
ENSG00000180777	ANKRD30B						U
ENSG00000162782	TDRD5						U
ENSG00000105825	TFPI2			U			U
ENSG00000007062	PROM1						U
ENSG00000013588	GPRC5A						U
ENSG00000115457	IGFBP2						U
ENSG00000164403	SHROOM1						U
ENSG00000112183	RBM24						U
ENSG00000163993	S100P						U
ENSG00000166920	C15orf48						U
ENSG00000117154	IGSF21						U
ENSG00000157214	STEAP2						U
ENSG00000124171	PARD6B						U
ENSG00000265766	CXADRP3						U
ENSG00000162078	ZG16B						U
ENSG00000138771	SHROOM3						U
ENSG00000144891	AGTR1						U
ENSG00000168350	DEGS2						U
ENSG00000070087	PFN2						U
ENSG00000147642	SYBU						U
ENSG00000169083	AR						U
ENSG00000078114	NEBL						U
ENSG00000155066	PROM2						U
ENSG00000123838	C4BPA			U			
ENSG00000112308	C6orf62			U			
ENSG00000167618	LAIR2			U			
ENSG00000262406	MMP12			U			
ENSG00000110195	FOLR1			U			
ENSG00000178878	APOLD1			U			
ENSG00000109321	AREG			U			
ENSG00000177606	JUN			U			
ENSG00000124193	SRSF6			U			
