# Supplementary material for: Molecular patterns of cancer colonisation in lymph nodes of breast cancer patients
Source: Breast Cancer Res. 2018 Nov 20;20:143. doi: 10.1186/s13058-018-1070-3 (PMC6247766; doi:10.1186/s13058-018-1070-3)
Supplement: Supplementary file 4 — Table S4. Pathway-based analysis of the differentially expressed genes across all the scenarios. (PDF 33 kb) [file 13058_2018_1070_MOESM4_ESM.pdf]

GO term	Description	Scenario 1	Scenario 2	Scenario 3	Scenario 4	Scenario 5	Scenario 6
G0:0001503	ossification	D	D				
G0:0007155	cell adhesion	U	D			D	
G0:0009888	tissue development	D		U			
G0:0022610	biological adhesion	U	D			D	
G0:0030198	extracellular matrix organization	D	D			U	
G0:0030574	collagen catabolic process	D					
G0:0032963	collagen metabolic process	D				U	
G0:0043062	extracellular structure organization	D	D			U	
G0:0044236	multicellular organism metabolic process	D				U	
G0:0044243	multicellular organismal catabolic process	D					
G0:0044259	multicellular organismal macromolecule metabolic process	D				U	
G0:0042127	regulation of cell proliferation	D	D				
G0:0008283	cell proliferation	D	D				
G0:0022617	extracellular matrix disassembly	D					
G0:0040011	locomotion	D	D			D	
G0:0048870	cell motility	D	D			D	
G0:0051674	localization of cell	D	D			D	
G0:0048646	anatomical structure formation involved in morphogenesis	D		U		U	
G0:0016477	cell migration	U	D			D	
G0:0006928	movement of cell or subcellular component	D	D			D	
G0:0072359	circulatory system development	D	D				
G0:0001501	skeletal system development	D					
G0:0009887	animal organ morphogenesis	D		U			
G0:0001944	vasculature development	D	D				
G0:0031214	biomineral tissue development	D					
G0:0072358	cardiovascular system development	D	D				
G0:2000026	regulation of multicellular organismal development	D	D	U			
G0:0001568	blood vessel development	D					
G0:0022603	regulation of anatomical structure morphogenesis	D	D				
G0:0009719	response to endogenous stimulus	D		U			
G0:0051270	regulation of cellular component movement	D					
G0:0071495	cellular response to endogenous stimulus	D					
G0:0030282	bone mineralization	D	D				
G0:0030155	regulation of cell adhesion	U	D				
G0:0070848	response to growth factor	D					
G0:0071363	cellular response to growth factor stimulus	D					
G0:2000145	regulation of cell motility	D					
G0:1901700	response to oxygen-containing compound	D					
G0:0051093	negative regulation of developmental process	D					
G0:0040012	regulation of locomotion	D					
G0:0016337	single organismal cell-cell adhesion	U				D	
G0:0045321	leukocyte activation	U					
G0:0046649	lymphocyte activation	U					
G0:0098609	cell-cell adhesion	U				D	
G0:0001775	cell activation	U					
G0:0098602	single organism cell adhesion	U				D	
G0:0006955	immune response	U					
G0:0002520	immune system development	U					
G0:0048534	hematopoietic or lymphoid organ development	U					
G0:0002682	regulation of immune system process	U					
G0:0030098	lymphocyte differentiation	U					
G0:0002521	leukocyte differentiation	U					
G0:0002684	positive regulation of immune system process	U					
G0:0030097	hemopoiesis	U					
G0:0002694	regulation of leukocyte activation	U					
G0:0050857	positive regulation of antigen receptor-mediated signaling pathway	U					
G0:0008219	cell death	U		U			
G0:0050865	regulation of cell activation	U					
G0:0012501	programmed cell death	U		U			
G0:0051249	regulation of lymphocyte activation	U					
G0:0006915	apoptotic process	U		U			
G0:0042113	B cell activation	U					
G0:0007159	leukocyte cell-cell adhesion	U					
G0:0042110	T cell activation	U					
G0:0070489	T cell aggregation	U					
G0:0071593	lymphocyte aggregation	U					
G0:0070486	leukocyte aggregation	U					
G0:0050851	antigen receptor-mediated signaling pathway	U					
G0:0043069	negative regulation of programmed cell death	U					
G0:0043067	regulation of programmed cell death	U					
G0:0022407	regulation of cell-cell adhesion	U					
G0:0050854	regulation of antigen receptor-mediated signaling pathway	U					
G0:0060548	negative regulation of cell death	U		U			
G0:0002696	positive regulation of leukocyte activation	U					
G0:0010941	regulation of cell death	U					
G0:0042981	regulation of apoptotic process	U					
G0:0060348	bone development		D				
G0:0008285	negative regulation of cell proliferation		D				
G0:0050673	epithelial cell proliferation		D				
G0:0043549	regulation of kinase activity		D				
G0:0045785	positive regulation of cell adhesion		D				
G0:0050678	regulation of epithelial cell proliferation		D				

G0:0045859	regulation of protein kinase activity		D				
G0:2000050	regulation of non-canonical Wnt signaling pathway		D				
G0:0051057	positive regulation of small GTPase mediated signal transduction		D				
G0:1901342	regulation of vasculature development		D				
G0:0006027	glycosaminoglycan catabolic process		D				
G0:0051050	positive regulation of transport		D				
G0:0042160	lipoprotein modification		D				
G0:0042161	lipoprotein oxidation		D				
G0:0060437	lung growth		D				
G0:0072223	metanephric glomerular mesangium development		D				
G0:0051094	positive regulation of developmental process		D				
G0:0014068	positive regulation of phosphatidylinositol 3-kinase signaling		D				
G0:0000280	nuclear division		U				
G0:0007059	chromosome segregation		U				
G0:0048285	organelle fission		U				
G0:0007067	mitotic nuclear division		U				
G0:1903047	mitotic cell cycle process		U				
G0:0051301	cell division		U				
G0:0051276	chromosome organization		U				
G0:0000278	mitotic cell cycle		U				
G0:0022402	cell cycle process		U				
G0:0098813	nuclear chromosome segregation		U				
G0:0000819	sister chromatid segregation		U				
G0:0007346	regulation of mitotic cell cycle		U				
G0:0007088	regulation of mitotic nuclear division		U				
G0:0007049	cell cycle		U				
G0:0051783	regulation of nuclear division		U				
G0:0051304	chromosome separation		U				
G0:0010564	regulation of cell cycle process		U				
G0:0030071	regulation of mitotic metaphase/anaphase transition		U				
G0:0051983	regulation of chromosome segregation		U				
G0:1902099	regulation of metaphase/anaphase transition of cell cycle		U				
G0:0007091	metaphase/anaphase transition of mitotic cell cycle		U				
G0:0010965	regulation of mitotic sister chromatid separation		U				
G0:0051726	regulation of cell cycle		U				
G0:0000070	mitotic sister chromatid segregation		U				
G0:0044784	metaphase/anaphase transition of cell cycle		U				
G0:0051306	mitotic sister chromatid separation		U				
G0:1902589	single-organism organelle organization		U				
G0:0033047	regulation of mitotic sister chromatid segregation		U				
G0:0033045	regulation of sister chromatid segregation		U				
G0:0044772	mitotic cell cycle phase transition		U				
G0:0033043	regulation of organelle organization		U				
G0:0044770	cell cycle phase transition		U				
G0:0033044	regulation of chromosome organization		U				
G0:0032101	regulation of response to external stimulus				D		
G0:0030193	regulation of blood coagulation				D		
G0:1900046	regulation of hemostasis				D		
G0:0050818	regulation of coagulation				D		
G0:0007596	blood coagulation				D	U	
G0:0050817	coagulation				D	U	
G0:0007599	hemostasis				D	U	
G0:0061041	regulation of wound healing				D		
G0:1903034	regulation of response to wounding				D		
G0:0050878	regulation of body fluid levels				D		
G0:0009617	response to bacterium				D		
G0:0042060	wound healing				D	U	
G0:0009605	response to external stimulus				D		
G0:0007229	integrin-mediated signaling pathway					D	
G0:0050900	leukocyte migration					U	
G0:0007160	cell-matrix adhesion					U	
G0:0034113	heterotypic cell-cell adhesion					D	
G0:0031589	cell-substrate adhesion					U	
G0:0050901	leukocyte tethering or rolling					D	
G0:0061756	leukocyte adhesion to vascular endothelial cell					D	
G0:0072678	T cell migration					D	
G0:0043113	receptor clustering					D	
G0:0043589	skin morphogenesis					U	
G0:0038065	collagen-activated signaling pathway					U	
G0:0001704	formation of primary germ layer					U	
G0:0070208	protein heterotrimerization					U	
G0:0007369	gastrulation					U	
G0:0009611	response to wounding					U	
G0:0071560	cellular response to transforming growth factor beta stimulus					U	
G0:0006929	substrate-dependent cell migration					U	
G0:0071559	response to transforming growth factor beta					U	
G0:0043588	skin development					U	
G0:0032964	collagen biosynthetic process					U	
G0:0030199	collagen fibril organization					U	
G0:0070206	protein trimerization					U	
G0:0035987	endodermal cell differentiation					U	
G0:1901698	response to nitrogen compound			U		U	
G0:0031346	positive regulation of cell projection organization					U	

G0:0001101	response to acid chemical					U	
G0:1901701	cellular response to oxygen-containing compound					U	
G0:0009790	embryo development					U	
G0:0001706	endoderm formation					U	
G0:0071230	cellular response to amino acid stimulus					U	
G0:0044364	killing of cells of other organism						D
G0:0031640	disruption of cells of other organism						D
G0:0045595	regulation of cell differentiation			U			
G0:0070372	regulation of ERK1 and ERK2 cascade			U			
G0:0070371	ERK1 and ERK2 cascade			U			
G0:0001932	regulation of protein phosphorylation			U			
G0:0043408	regulation of MAPK cascade			U			
G0:0042325	regulation of phosphorylation			U			
G0:0051591	response to cAMP			U			
G0:0010243	response to organonitrogen compound			U			
G0:0008284	positive regulation of cell proliferation			U			
G0:0009725	response to hormone			U			
G0:0019220	regulation of phosphate metabolic process			U			
G0:0000165	MAPK cascade			U			
G0:0051174	regulation of phosphorus metabolic process			U			
G0:0042542	response to hydrogen peroxide			U			
G0:0001934	positive regulation of protein phosphorylation			U			
G0:0032355	response to estradiol			U			
G0:0031399	regulation of protein modification process			U			
G0:0023014	signal transduction by protein phosphorylation			U			
G0:0001525	angiogenesis			U			
G0:0042327	positive regulation of phosphorylation			U			
G0:0046683	response to organophosphorus			U			
G0:0014074	response to purine-containing compound			U			
G0:0006468	protein phosphorylation			U			
G0:0010562	positive regulation of phosphorus metabolic process			U			
G0:0045937	positive regulation of phosphate metabolic process			U			
G0:0031960	response to corticosteroid			U			
G0:0048514	blood vessel morphogenesis			U			
G0:0060429	epithelium development			U			
G0:0009628	response to abiotic stimulus			U			
G0:0031401	positive regulation of protein modification process			U			
