# Supplementary material for: Molecular patterns of cancer colonisation in lymph nodes of breast cancer patients
Source: Breast Cancer Res. 2018 Nov 20;20:143. doi: 10.1186/s13058-018-1070-3 (PMC6247766; doi:10.1186/s13058-018-1070-3)
Supplement: Supplementary file 5 — Table S5. Differentially expressed genes representing specific immune cell populations across all the scenarios. (PDF 21 kb) [file 13058_2018_1070_MOESM5_ESM.pdf]

Ensembl ID	Gene Name	Cell Type	Scenario 1	Scenario 2	Scenario 3	Scenario 4	Scenario 5	Scenario 6
ENSG00000121966	CXCR4	T cells	U		U			
ENSG00000115414	FN1	DC	D				U	
ENSG00000139626	ITGB7	Th1					D	
ENSG00000026508	CD44	MDSC					D	
ENSG00000115232	ITGA4	Th1					D	
ENSG00000132185	FCRLA	Immature B cells	U					D
ENSG00000196092	PAX5	Activated B cells	U					D
ENSG00000213658	LAT	T cells	U					D
ENSG00000134443	GRP	iDC	D					D
ENSG00000069482	GAL	Activated CD8						D
ENSG00000105205	CLC	Mast cells						D
ENSG00000124469	CEACAM8							D
ENSG00000254415	SIGLEC14	Mast cells						D
ENSG00000104921	FCER2	MDSC						D
ENSG00000167483	FAM129C	Immature B cells						D
ENSG00000183691	NOG	MDSC						D
ENSG00000137101	CD72	Treg						D
ENSG00000012124	CD22	Immature B cells						D
ENSG00000142512	SIGLEC10	Th2						D
ENSG00000124334	IL9R	Treg						D
ENSG00000176884	GRIN1	NK56 dim						D
ENSG00000131196	NFATC1	T cells						D
ENSG00000120539	MASTL	Activated CD4						D
ENSG00000163564	PYHIN1	Central memory CD8						D
ENSG00000134256	CD101	NKT						D
ENSG00000223865	HLA-DPB1	Effector memory CD8	U					
ENSG00000137509	PRCP	mDC	U					
ENSG00000102265	TIMP1	Mast cells	U					
ENSG00000081237	PTPRC	Th1	U					
ENSG00000227507	LTB	Th1	U					
ENSG00000188404	SELL	Cytotoxic cells	U					
ENSG00000090104	RGS1	Central memory CD8	U					
ENSG00000023445	BIRC3	Effector memory CD8	U					
ENSG00000169508	GPR183	Central memory CD8	U					
ENSG00000172724	CCL19	Treg	U					
ENSG00000141293	SKAP1	Th2	U					
ENSG00000169442	CD52	Th1	U					
ENSG00000137077	CCL21	Activated B cells	U					
ENSG00000083454	P2RX5	Th1	U					
ENSG00000153064	BANK1	Immature B cells	U					
ENSG00000163534	FCRL1	Immature B cells	U					
ENSG00000009790	TRAF3IP3	Activated B cells	U					
ENSG00000125245	GPR18	TFH	U					
ENSG00000035720	STAP1	Immature B cells	U					
ENSG00000163519	TRAT1	Th1	U					
ENSG00000162894	FCMR		U					
ENSG00000241106	HLA-DOB	Immature B cells	U					
ENSG00000185811	IKZF1	MDSC	U					
ENSG00000168685	IL7R	Th1	U					
ENSG00000164691	TAGAP	Immature B cells	U					
ENSG00000160856	FCRL3	Immature B cells	U					
ENSG00000078081	LAMP3	Th2	U					
ENSG00000182866	LCK	T cells	U					
ENSG00000126353	CCR7	TFH	U					
ENSG00000162692	VCAM1	DC	U					
ENSG00000167286	CD3D	T cells	U					
ENSG00000172005	MAL	Central memory CD4	U					
ENSG00000197943	PLCG2	NK	U					
ENSG00000140968	IRF8	T cells	U					
ENSG00000134954	ETS1	Immature B cells	U					
ENSG00000143119	CD53	Th1	U					
ENSG00000112149	CD83	TFH	U					
ENSG00000110031	LPXN	iDC	U					
ENSG00000163751	CPA3	Mast cells	D					
ENSG00000162493	PDPN	DC	D					
ENSG00000135914	HTR2B	Th2	D					
ENSG00000158477	CD1A	T cells	D					
ENSG00000204592	HLA-E	NK56 dim	D					
ENSG00000136235	GPNMB	TGD	D					
ENSG00000104213	PDGFRL	DC	D					
ENSG00000123610	TNFAIP6	Mast cells	D					
ENSG00000124813	RUNX2	Memory B cells	D					
ENSG00000203747	FCGR3A	Effector memory CD8	D					
ENSG00000122862	SRGN	Monocytes	D					
ENSG00000099860	GADD45B	Treg	D					
ENSG00000134363	FST	Mast cells	D					
ENSG00000131981	LGALS3	MDSC	D					
ENSG00000164949	GEM	Treg	D					
ENSG00000118785	SPP1	TGD	D					
ENSG00000117322	CR2	MDSC	B					
ENSG00000117091	CD48	Th1	B					
ENSG00000164109	MAD2L1	Activated CD4		U				
ENSG00000115415	STAT1	T cells		U				
ENSG00000138160	KIF11	Activated CD4		U				
ENSG00000011426	ANLN	Activated CD4		U				
ENSG00000126787	DLGAP5	Activated CD4		U				
ENSG00000120738	EGR1	Eosinophil		D				
ENSG00000144642	RBMS3	Th2		D				
ENSG00000174059	CD34	Treg		D				
ENSG00000017427	IGF1	Macrophages		D				
ENSG00000134138	MEIS2	Mast cells		D				
ENSG00000092009	CMA1	Mast cells		D				
ENSG00000164741	DLC1	Th2		D				
